# Trait-Based Community Assembly along an Elevational Gradient in Subalpine Forests: Quantifying the Roles of Environmental Factors in Inter- and Intraspecific Variability

**DOI:** 10.1371/journal.pone.0155749

**Published:** 2016-05-18

**Authors:** Ya-Huang Luo, Jie Liu, Shao-Lin Tan, Marc William Cadotte, Yue-Hua Wang, Kun Xu, De-Zhu Li, Lian-Ming Gao

**Affiliations:** 1 School of Life Sciences, Yunnan University, Kunming, China; 2 Key Laboratory for Plant Diversity and Biogeography of East Asia, Kunming Institute of Botany, Chinese Academy of Sciences, Kunming, China; 3 Germplasm Bank of Wild Species in Southwest China, Kunming Institute of Botany, Chinese Academy of Sciences, Kunming, China; 4 University of Chinese Academy of Sciences, Beijing, China; 5 Biological Sciences, University of Toronto-Scarborough & Ecology and Evolutionary Biology, University of Toronto, Toronto, Ontario, Canada; 6 State Key Laboratory of Biocontrol, Key Laboratory of Biodiversity Dynamics and Conservation of Guangdong, Higher Education Institutes, College of Ecology and Evolution, Sun Yat-sen University, Guangzhou, China; 7 Lijiang Forest Ecosystem Research Station, Kunming Instituted of Botany, Chinese Academy of Sciences, Lijiang, China; USDA-ARS, UNITED STATES

## Abstract

Understanding how communities respond to environmental variation is a central goal in ecology. Plant communities respond to environmental gradients via intraspecific and/or interspecific variation in plant functional traits. However, the relative contribution of these two responses to environmental factors remains poorly tested. We measured six functional traits (height, leaf thickness, specific leaf area (SLA), leaf carbon concentration (LCC), leaf nitrogen concentration (LNC) and leaf phosphorus concentration (LPC)) for 55 tree species occurring at five elevations across a 1200 m elevational gradient of subalpine forests in Yulong Mountain, Southwest China. We examined the relative contribution of interspecific and intraspecific traits variability based on community weighted mean trait values and functional diversity, and tested how different components of trait variation respond to different environmental axes (climate and soil variables). Species turnover explained the largest amount of variation in leaf morphological traits (leaf thickness and SLA) across the elevational gradient. However, intraspecific variability explained a large amount of variation (49.3%–76.3%) in three other traits (height, LNC and LPC) despite high levels of species turnover. The detection of limiting similarity in community assembly was improved when accounting for both intraspecific and interspecific variability. Different components of trait variation respond to different environmental axes, especially soil water content and climatic variables. Our results indicate that intraspecific variation is critical for understanding community assembly and evaluating community response to environmental change.

## Introduction

Plant functional traits are linked to the ecological strategies of species and directly influence species interactions, making them fundamental drivers of community assembly [1–3]. Most current trait-based studies in community ecology focus on analyses of trait dispersion among species in order to infer environmental filtering and limiting similarity [4–14]. Environmental filtering results in a relatively small range of trait values (i.e., under-dispersed or clustered) occurring in specific environmental conditions [9, 14], but other processes (such as competition) could also select species with similar traits [15]. Alternatively, limiting similarity occurs when competition excludes species with trait values that are too similar to other species that have a competitive advantage (e.g., higher fitness) [16–18], resulting in communities that contain dissimilar species (i.e., over-dispersed). Thus, the composition of any given community likely results from a combination of environment filtering and limiting similarity [9, 19]. In addition to these deterministic processes, neutral dynamics randomly structure a community’s functional traits and community composition [20].

The mechanisms of community assembly described above are often used to understand plant community responses to environmental gradients and are most commonly studied at the species level [4, 21]. However, intraspecific trait variability is known to play a fundamental role in plant community responses to environmental change and community assembly [19, 22–31]. Recent studies indicate that accounting for intraspecific trait variation could improve the detection of non-random patterns in community assembly [7, 19, 32]. For example, Siefert (2012) reported statistical signatures of environmental filtering are stronger when intraspecific trait variation is taken into account [32]. Furthermore, intraspecific functional trait variation might reflect underlying environmental gradients or stress. For instance, short-term events such as extreme droughts likely induce community functional responses mainly through intraspecific variability caused by plastic phenotypic responses [29].

The relative contribution of intraspecific trait variation to shifts in community-average trait values along environmental gradients reflects the importance of within-species trait responses to environmental stress and measuring this variation can help the detection and interpretation of assembly patterns [23, 27, 33]. For example, variation in community composition across environmental gradients might be lower than expected partly because of within-species trait responses to environmental gradients [29, 34]. However, the relative contribution of intraspecific variation and species turnover to the total trait variation along environmental gradients is poorly understood [35]. Further complicating our understanding of how intraspecific variation is influenced by environmental gradients is that the explanatory percentage of intraspecific variation to trait variation varies among studies, and results might be dependent on the functional traits measured, spatial scale of observation, and the study habitat type, for example, tropical forests [11, 36], subtropical forests [37], and temperate grasslands [13, 19, 24, 29].

Strong environmental gradients provide a unique opportunity to examine the relative contributions of inter- and intraspecific trait variation. One of the most commonly employed natural gradients is vegetation change along elevation gradients, with variation driven by underlying edaphic and climatic factors [35]. Our study site is in the subalpine system of the Hengduan Mountains of southwest China with dramatic variation in topography, habitat and climate, and the region is considered to be extremely sensitive to climate change and land-use shifts [38–40]. The response of plant communities across such stark environmental gradients provides an ideal setting for studying functional diversity and community assembly.

We observed that plant functional traits, such as height and leaf area varied among forest communities along the elevational gradient at Yulong Mountain. To quantify the importance of intraspecific trait variation in the assembly of subalpine forest communities, and to further test how inter- and intraspecific variation respond to different abiotic variables, we examined functional traits and environmental variables to address the following three questions: 1) What is the relative contribution of interspecific and intraspecific variation to a shift in the community weighted mean (CWM) of functional traits along the elevational gradient? 2) Whether the detection of nonrandom community trait patterns (i.e., over-dispersed vs. clustered) could be improved by accounting for both intra- and interspecific trait variability compared to interspecific trait variability only? 3) How do inter- and intraspecific trait variation respond to different environmental axes under consideration? To address these questions, we measured six functional traits (plant height and five leaf functional traits) of tree species collected from subalpine forest plots at five elevations of the Yulong Mountains.

## Materials and Methods

### Ethics Statement

Permission for field sampling was obtained from the Lijiang Alpine Botanical Garden, Kunming Institute of Botany. None of the species sampled in this study are endangered or protected species.

### Study area

The study site (27°00’12” N, 100° 10’ 50” E) is located at the southern edge of Yulong Mountain in Lijiang, Yunnan province, China. Yulong Mountain was recognized as the southernmost mountain glacier in the northern hemisphere and extremely sensitive to climate change. It belongs to the Henduan Mountains region and is located in the southeastern margin of the Qinghai-Tibet Plateau, which is known as the center of “Mountains of Southwest China” and as a global biodiversity hotspot [41–43]. At Yulong Mountain, the mean annual temperature is 12.8°C and the annual precipitation is 935 mm [44], with a dry season from November to May and rainy season from June to October caused by the southwest monsoon carrying moisture from the Indian Ocean. The study area is in a protected area of the Lijiang Alpine Botanical Garden of the Kunming Institute of Botany, Chinese Academy of Sciences, and has an elevation range from 2650 m to 3850 m.

The vegetation zonation is obvious along elevational gradient in Yulong Mountain. At the lowest elevation (2650 m), the forest vegetation is dominated by *Pinus armandii*. At 2950 m, the forest vegetation shifts and is dominated by the evergreen conifers *P*. *armandii* and *Pinus yunnanensis*, and the sclerophyllous evergreen broad-leaved *Quercus spinosa*, forming a mixed coniferous / sclerophyllous broad-leaved forest. At 3250 m, the forest vegetation changes again and is dominated by *Quercus guyavifolia* and *P*. *yunnanensis*. At the highest elevation (3850 m) the forest composition is dominated by *Quercus aquifolioides*, *Abies georgei* and *Rhododendron rubiginosum*.

### Field survey

**Fig 1:**
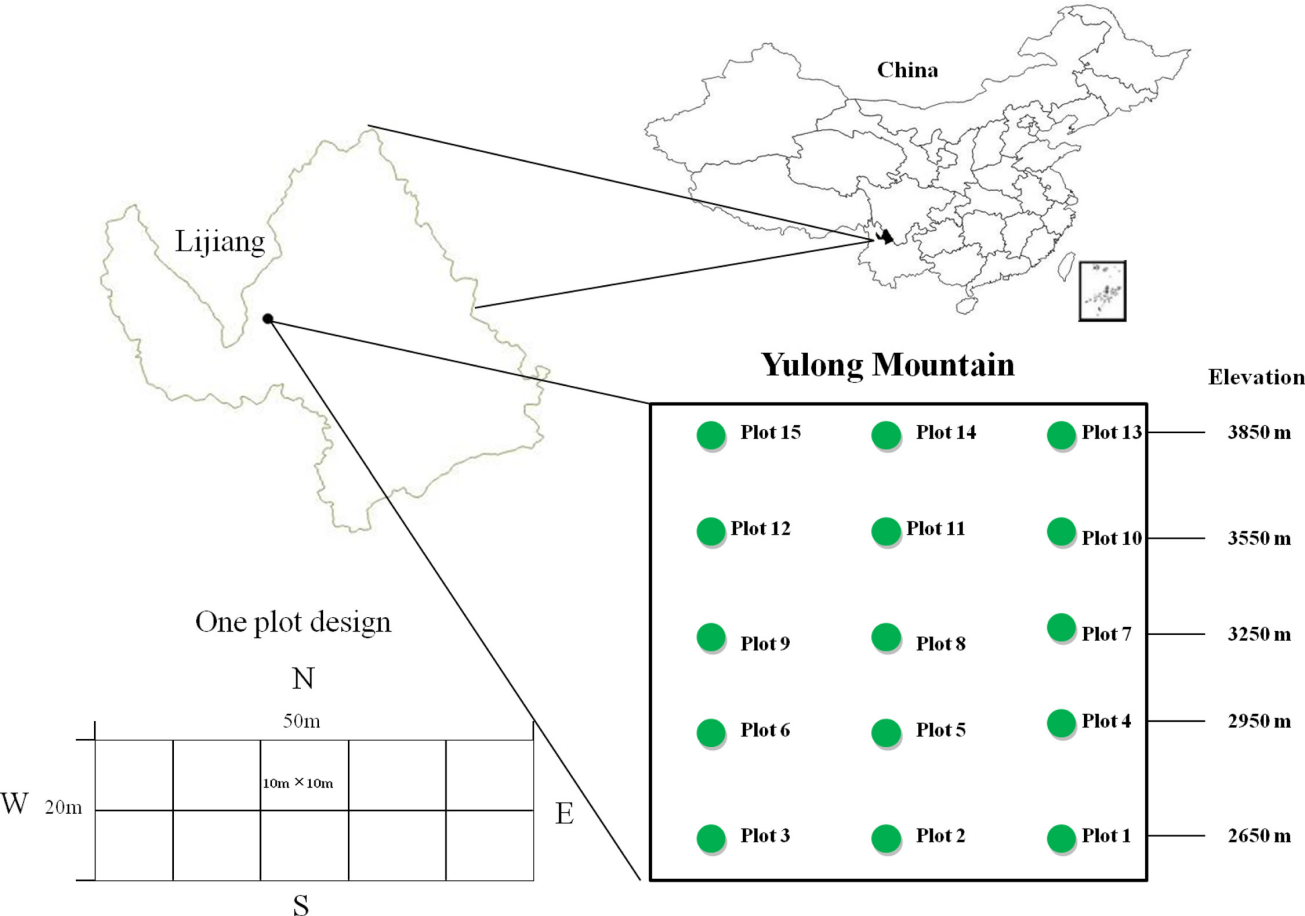
Map of the study site in Yulong Mountain, Lijiang, Yunnan, China and the plot design, 15 plots (green dot) are shown along the elevational gradient. Data obtained from the National Fundamental Geographic Information System (NFGIS, http://ngcc.sbsm.gov.cn/), then edited using ArcGIS 10.2 (ESRI, Redlands, CA, USA).

A total of 15 forest plots of 0.1 ha (20 m × 50 m) were established at five elevations (2650 m, 2950 m, 3250 m, 3550 m and 3850 m; three plots at each elevation) from May to August 2013 (Fig 1).The five elevations were selected to cover all vegetation types, and three replications at each elevation were randomly arranged with at least 100 m apart from each other (Fig 1). Each plot was divided into ten subplots (10 m ☓10 m). All individuals of woody species with DBH≥1cm were recorded and identified to species by taxonomic specialists for accurate identification. In total, 4782 trees of 55 species (eight gymnosperms and 47 angiosperms) were recorded in the plots (S1 Table). A voucher specimen for each species in every plot was collected and deposited at the herbaria of Kunming Institute of Botany (KUN), CAS, Yunnan, China.

### Measurements of functional traits

We measured six functional traits including plant height, leaf thickness, specific leaf area (SLA), leaf carbon concentration (LCC), leaf nitrogen concentration (LNC) and leaf phosphorus concentration (LPC). These functional traits are known to be sensitive to soil and climatic variables [23, 29, 45]. Plant height (maximum height of the canopy) was measured for each species by using Vertex IV hypsometer (Haglöf Sweden AB, Långsele, Sweden). Leaf traits were measured from June to August 2013, the period of peak leaf growth, on randomly collected full sun exposed canopy leaves from adult plants that showed no obvious symptom of pathogen or physical damage, with the petiole and rachis considered part of the leaf [46]. We sampled 14 to 20 leaves from seven to ten individuals (two leaves from each individual) for each species in each plot. For rare species, leaves were collected from outside of the plots but not more than 20 m to the plot edge. In total, 2964 leaves were sampled. An electronic digital caliper was used to measure leaf thickness (mm) at the center of the lamina by avoiding major leaf veins. Each broad leaf was scanned with a CanonScan LiDE 210 (Canon Inc., Tokyo, Japan) and the leaf area calculated using Image J [47]. For needle-shaped leaves, the leaf area was calculated following the method of Turner *et al*. [48], which is based on the needle shape and volume, number of needles and mean needle length. SLA was calculated as leaf area divided by leaf dry mass (after the leaf was dried to a constant weight at 70°C).Leaf chemical traits were measured using Kjeldahl analysis. LCC and LNC were measured with a Vario MAX CN elemental analyzer (Elementar Analysensysteme GmbH, Hanau, Germany), and LPC was analyzed with an iCAP6300 elemental analyzer (Thermo Fisher Scientific, Waltham, MA, USA).

### Environmental data collection

In order to obtain average soil characteristics within each plot, soil samples from four different depth layers (0–10 cm, 10–20 cm, 20–30 cm, 30–50 cm) were collected from five profiles per plot. For each layer in each plot, soil samples were pooled, homogenized, air-dried and sieved (2-mm) for further analyses. Six soil variables were measured including pH, total carbon, total nitrogen, total phosphorus, available phosphorus and available potassium contents. Since previous work showed that functional traits were associated with mean annual temperature, annual precipitation and soil water content indices [1, 7], we collected climatic data for precipitation, air temperature and soil temperature (at 10cm depth) using HOBO RG3-M, HOBO Pro v2 and HOBO Tidbit v2 respectively (Onset Computer Corporation, Bourne, MA, USA) from June 2013 to June 2014 at each elevation in this study. Based on these data, we derived three climatic variables, annual precipitation (AP), air mean annual temperature (AMAT), and soil mean annual temperature (SMAT). We measured the soil water content of the top 0–15cm soil layer on 24–25th March, 2015 using MiniTrase Kit (Soil Moisture Equipment Corp., Santa Barbara, CA, USA) following Cornwell and Ackerly [7]. We collected the soil water content data for 30 cores (three cores within each subplot) located randomly within each plot. Because of logistical constraints, we sampled plant functional traits in 2013 but measured soil moisture in 2015. This was not ideal, however, climate conditions were similar according to environmental records from a nearby field station. Furthermore, we assume that relative differences in soil moisture across the elevation gradient would be relatively consistent across years.

### Data analysis

Several analyses were required to test our hypotheses about the relative importance of intraspecific variability to CWMs of traits, patterns of community assembly, and how intra- and interspecific variation respond to environmental gradients. Specifically, our analyses move from species turnover to measuring trait variation to tests of community assembly.

#### Species diversity and composition

To estimate species diversity along the elevational gradient, species richness, evenness and both the Shannon and Simpson diversity indices were calculated for each plot. The Sorensen pair-wise dissimilarity index of species composition was calculated to quantify species turnover among plots. The relationship between Sorensen pair-wise dissimilarity and plot elevation difference was assessed using a Mantel test.

#### Partitioning trait variability

To examine functional trait composition, the community weighted mean (CWM) was calculated for the six functional traits. Since large trait values have a greater influence on the arithmetic mean making it more prone to sampling error [49], all functional traits were log transformed before analysis. CWM represents the mean trait value of a community considering the relative abundance of each species at a specific site. Three CWM values were calculated following Lepš *et al*.’s method [22]. We calculated two CWMs, a ‘Specific average’ that captures trait values within plots, and a ‘Fixed average’ that uses species’ means trait values across all plots. The ‘Specific average’ was calculated using trait values measured in each plot as: $\text{Specific average}=\;\sum_{\text{i}=1}^{\text{n}}{\text{A}}_{\text{ij}}{\text{T}}_{\text{ij}}$ where A_ij_ is the abundance of species i in plot j, and T_ij_ is the mean trait value of species i measured in plot j, n is the number of species in the site. ‘Fixed average’ was calculated as: $\text{Fixed average}=\;\sum_{\text{i}=1}^{\text{n}}{\text{A}}_{\text{ij}}{\text{T}}_{\text{i}}$ where T_i_ is the mean trait value of species i across all plots. To estimate intraspecific trait variability we simply subtracted the Fixed average from the Specific average: Intraspecific variability effect = Specific average–Fixed average.

To compare the relative importance of intra- and interspecific variability, we partitioned inter- and intraspecific trait variability effects on weighted plot-level traits values among plots also following the approach by Lepš *et al*. [22]. Specifically, the total sum of squares of species trait variance for all plots (SS_specific_) was decomposed into ‘fixed’ (SS_fixed_), ‘intraspecific’ (SS_intraspecific_) and ‘covariation’ (SS_cov_) effects, thus SS _specific_ = SS_fixed_ + SS_intraspecific_ + SS_cov_. For each plot and trait, ‘specific’ community mean trait values using species trait values as measured on each plot (which includes both inter- and intraspecific effects), and ‘fixed’ community mean trait values using species trait values averaged over all plots (which removes the intraspecific variability effect), ‘intraspecific’ plot means for each were calculated as the difference between ‘specific’ and ‘fixed’ plot mean trait values [22, 23, 31, 50]. Positive or negative covariation values indicate that the relationship between ‘fixed’ and ‘intraspecific’ effects reinforce or oppose each other, respectively.

#### Community assembly tests

**Fig 2:**
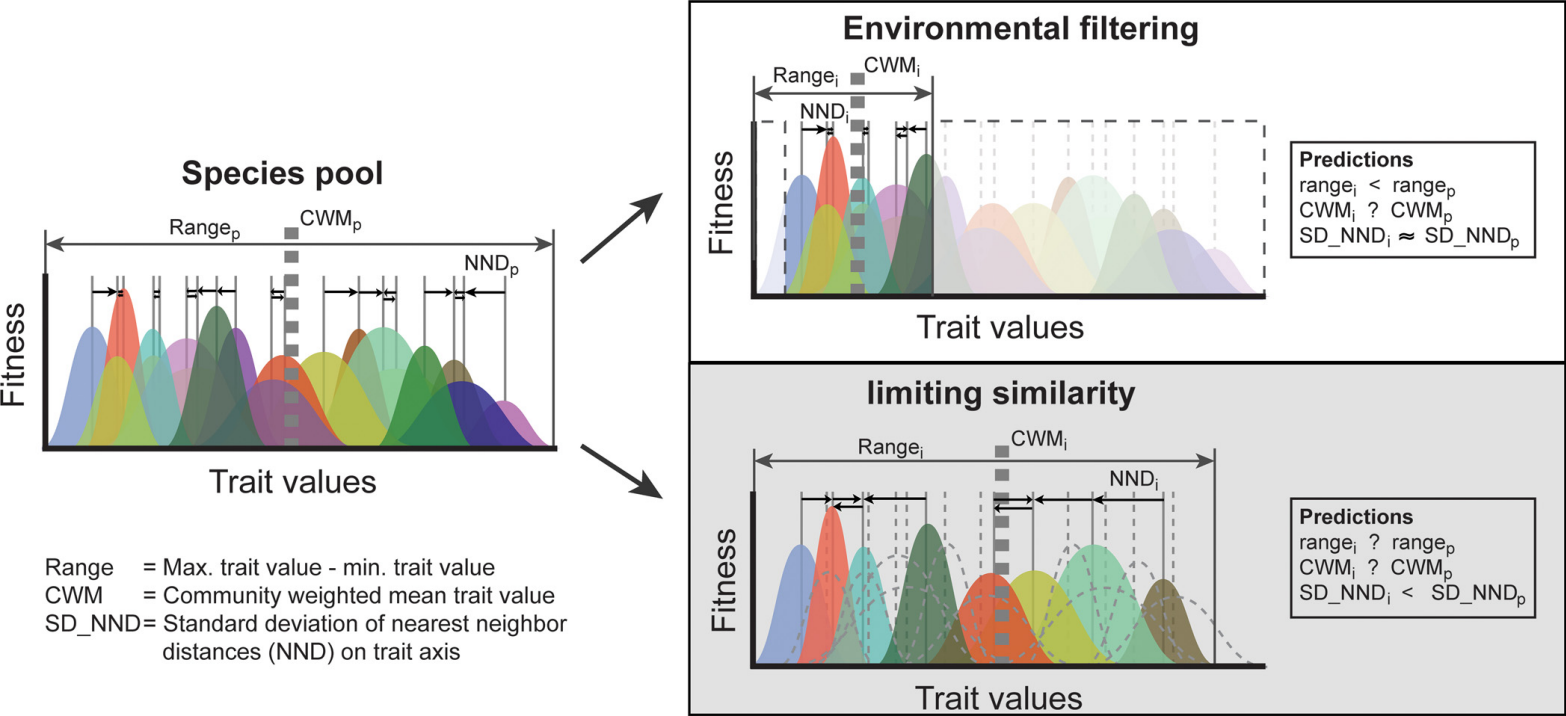
An illustration of incorporating intraspecific variation in tests of trait-based community assembly. In our analyses, we use three distinct trait based measures that should reflect underlying assembly mechanisms. Differences between the species pool (p) and sampled community (i) in the trait range, community weighted mean, and the standard deviation of nearest-neighbor distances reflect the dominant mechanism. For environmental filtering, the trait range should be reduced as a subset of traits is selected. Under limiting similarity, neighboring trees have divergent traits (i.e. increased trait distance). Furthermore, the standard deviation of nearest-neighbor distances in the sampled community should be less than the pool. Specifically, more even trait distributions among co-occurring species could be detected in observed communities compared to random expectations. Whether the CWM traits change depends on if trait selection is biased away from the pool mean. Intraspecific variation is shown as colored distributions, and non-random selection of intraspecific variation could strengthen assembly patterns (e.g., plot individuals more divergent under limiting similarity than predicted by species means).

*Tests for environmental filtering*. To test the effect of environmental filtering on community assembly, a null model approach was used to test whether the observed trait metrics differ from random (Fig 2). If environmental filtering is occurring at the plot scale, the range of trait values in observed communities would be smaller than the null expectation. Environmental filtering may also shift the community weighted mean of the plot trait distribution relative to the null expectation [9, 10, 19, 51].

In order to incorporate intraspecific trait variability effect into the detection of environmental filtering, separate tests were conducted that accounted for: 1) interspecific trait variation only, using species mean trait values, 2) including inter- and intraspecific trait variation by using plot-specific species mean trait values. For the null model, species were drawn randomly from the species pool (55 species), weighted by frequency of occurrence across 15 plots [51]. The test statistics of trait metrics are highly correlated with species richness, so randomizations were constrained to observed plot richness [7, 32]. For the null model used with plot-specific species mean trait data, species were randomly drawn from the species pool and then randomly assigned to one of its plot-specific mean trait values [19, 32]. Each randomization procedure was run 999 times.

We used the randomizations to determine if trait CWM and range were significantly nonrandom in each plot (Fig 2). The observed CWM or range value was considered significant when it was outside the lower or upper 95% percentile of the null model. We tested if the trait range changed along elevational gradients. Further, to understand how functional composition (CWM) and range varies along the elevational gradient, simple linear regressions were used to test for a relationship between CWM (or range) and elevation.

To evaluate the deviation of observed trait metrics from mean expected values for each trait across plots, Wilcoxon- signed rank tests were applied. Since we did not expect a directional shift (Fig 2), CWM tests were two tailed. Meanwhile, we expected the observed range of trait values to be smaller than the null expectation for testing environmental filtering (Fig 2), thus range tests were one-tailed.

*Tests for limiting similarity*. To detect limiting similarity, we also used a null model method to test whether the observed trait metrics deviated from null expectations. Assuming limiting similarity is occurring, we anticipate high levels of trait dispersion among neighboring trees (intra and/or interspecific variation) (Fig 2). This will result in neighboring trees consistently having divergent traits (i.e. increased trait distance) (Fig 2). Furthermore, under limiting similarity, we predict that the standard deviation of nearest-neighbor distances (SD_NND) trait values will be smaller than the null model as species become evenly positioned in trait space [9, 10, 19].

To examine the effect of intraspecific trait variability on the detection of limiting similarity, separate tests that accounted for both intra- and interspecific trait variability and interspecific variability only were conducted as well. For the null model, the same approach used in detecting environmental filtering was also applied in detecting limiting similarity. In addition, to account for the influence of dispersal limitation in high elevation subalpine forests, we also performed a null model that constrained the selection of species from each specific elevation.

Standard deviation of nearest-neighbor distances (SD_NND) of trait value was calculated to evaluate the role of limiting similarity in community assembly for each trait in each plot. We compared whether SD_NND values changed with increasing species richness, and whether the observed SD_NND values were significantly smaller than random values.

To evaluate the deviation of observed SD_NND values from mean expected values for each trait across all plots, Wilcoxon- signed rank tests were also applied. Because limiting similarity is expected to shift SD_NND of the observed trait values below the null expectation (Fig 2). Standard deviation of nearest-neighbor distances (SD_NND) tests were one-tailed.

#### Quantifying the roles of different environmental axes in trait variability

A principal component analysis (PCA) was used to reduce the number of soil variables, and we selected the first three orthogonal axes of the PCA, which combined explained 88.81% of soil data variance. The first principal component axis (soilPC1) is negatively correlated with total phosphorus content and positively correlated with available potassium content. The second principal component (soilPC2) is negatively correlated with total nitrogen and total carbon contents, and positively correlated with soil pH. Finally, the third principal component (soilPC3) is negatively correlated with available potassium content (S1 Fig). Guassian-distributed generalized linear model regressions were used to determine the response of community weighted mean trait values to single environmental variables including: soil properties, March soil water content, annual precipitation (AP), air mean annual temperature (AMAT) and soil mean annual temperature (SMAT). Guassian-distributed multiple general linear models were also performed to select multiple combined edaphic and climatic variables that could best predict community weighted mean trait values based on lowest Akaike information criteria (AIC). Finally, the approach of Lepš et al. [22] was also used to partition the interspecific and intraspecific variance explained by each soil and climatic variable. Briefly, total sum of squares (SS_specific_) of the plot-level trait variance related to an environmental variable (here soil water content, AP, AMAT, SMAT, soilPC1, soilPC2 and soilPC3) into ‘fixed’ (SS_fixed_), ‘intraspecific’ (SS_intraspecific_) and ‘covariation’ (SS_cov_) effects.

All analyses above were performed in R (R 3.1.3 version) [52].

## Results

### Species diversity and turnover

Species richness, evenness and Shannon, Simpson diversity exhibited a unimodal relationship with the elevational gradient, with maximal values at 2965 m (S2 Table). The plot dissimilarity values were positively correlated with elevational difference (r = 0.81, *P* = 0.001). In other words, greater compositional dissimilarity was observed for plots further apart in elevation. Even within the same elevation strata, some of the plots had dissimilar species composition as measured by the high Sorensen pair-wise dissimilarities (S2 Fig).

### Relative importance of intraspecific variability

Intraspecific variability contributed a greater proportion to the functional shift than interspecific variability for both height and leaf chemical traits, while interspecific variability was more important for leaf morphological traits. For SLA, the CWM trait variation was almost completely generated by interspecific variation, which accounted for 89.0% of the total variation of SLA, and there was positive covariation between interspecific and intraspecific variation among the plots (Table 1). The contribution of interspecific variation was greater than intraspecific variation for leaf thickness (66.2% vs. 24.8%) and LCC (52.2% vs. 19.7%), but they covaried positively. However, intraspecific variability accounted for 76.3%, 49.3% and 68.7% of the community weighted mean height, LNC and LPC variation respectively, with only plant height having a negative covariation (-0.402) between interspecific and intraspecific variation (Table 1).

**Table 1:** The proportion of interspecific variation, intraspecific variation and covariation effects contributing to the variance in community weighted mean trait values among plots.

Traits	Interspecific variation effect	Intraspecific variation effect	Covariation effect
Height	0.639	0.763	-0.402
Leaf thickness	0.662	0.248	0.090
SLA	0.890	0.023	0.087
LCC	0.522	0.197	0.281
LNC	0.317	0.493	0.190
LPC	0.203	0.687	0.110

### Effect of intraspecific variability on environmental filtering

Environmental filtering was not detected for most traits across all plots, and intraspecific variability did not contribute to the detection of environmental filtering (Table 2). The only exception we found was when we accounted for intraspecific variability in the CWM of leaf thickness, which significantly deviated from null expectations across all plots (*P* = 0.03), and the range of LCC, which was significantly smaller than expectations when both intra- and interspecific variability was included (*P* = 0.015) or when using interspecific variability alone (*P* = 0.025). For other traits, the CWM did not deviate from null models, and the range was not smaller than null expectations (Table 2).

**Table 2:** Results of environmental filtering and limiting similarity tests using a null model approach. Wilcoxon signed-rank test comparing observed community weighted mean (CWM), range and standard deviation of nearest-neighbor distances (SD_NND) of traits to the means of the null expectation across all plots (n = 15), by considering intraspecific variability or not. Restricted SD_NND is the result of null model that constrained the species selection to each specific elevation. Two-tailed tests were used to the community weighted mean, and one-tailed tests were used to the range and SD_NND respectively, values in bold are significant (*P*< 0.05). The numbers in brackets refer to the number of plots in which the observed trait metric was significantly more or less than the null expectation.

		CWM			Range		SD_NND		Restricted SD_NND	
Traits	Source of variability	No. of plots with observation > expectation (out of 15)	No. of plots with observation < expectation (out of 15)	Wilcoxon *P*	No. of plots with observation < expectation (out of 15)	Wilcoxon *P*	No. of plots with observation < expectation (out of 15)	Wilcoxon *P*	No. of plots with observation < expectation (out of 15)	Wilcoxon *P*
Height	Interspecific	11 (0)	4 (1)	0.169	8 (2)	0.445	12 (0)	0.115	10 (0)	0.104
	Inter- and intraspecific	10 (3)	5 (0)	0.121	6 (1)	0.577	12 (3)	**0.021**	12 (3)	**0.003**
Leaf thickness	Interspecific	11 (1)	4 (1)	0.107	8 (1)	0.18	13 (1)	**0.001**	12 (0)	**0.002**
	Inter- and intraspecific	12 (2)	3 (1)	**0.03**	8 (2)	0.18	12 (0)	**0.018**	11(5)	**0.028**
SLA	Interspecific	7 (0)	8 (4)	0.303	5 (3)	0.467	12 (4)	**0.042**	13 (0)	**0.02**
	Inter- and intraspecific	7 (0)	8 (4)	0.208	4 (0)	0.835	15 (11)	**<0.001**	15 (11)	**<0.001**
LCC	Interspecific	9 (0)	6 (1)	0.712	10 (1)	**0.025**	11 (1)	0.084	9 (0)	0.489
	Inter- and intraspecific	7 (2)	8 (2)	0.804	10 (2)	**0.015**	12 (0)	**0.009**	10 (5)	**0.009**
LNC	Interspecific	7 (0)	8 (0)	0.121	9 (0)	0.084	13 (4)	**0.037**	10 (0)	0.195
	Inter- and intraspecific	6 (0)	9 (0)	0.151	7 (0)	0.511	12 (1)	0.126	12 (5)	0.06
LPC	Interspecific	5 (0)	10 (0)	0.188	8 (5)	0.151	11 (1)	**0.024**	11 (0)	**0.011**
	Inter- and intraspecific	7 (0)	8 (0)	0.489	10 (5)	0.076	11 (4)	**0.011**	14 (6)	**<0.001**

We explored whether accounting for intraspecific variability improved the detection of environmental filtering for each plot. First, we found that some traits varied with elevation. For example, CWM of SLA (r^2^ = 0.505, *P* = 0.003) and LCC (r^2^ = 0.606, *P* < 0.001) significantly increased with increasing elevation, and accounting for intraspecific variability did not improve these relationships: CWM of SLA (r^2^ = 0.487, *P* = 0.004), LCC (r^2^ = 0.687, *P* < 0.001) (S3 Fig). The range of height and SLA values significantly decreased with elevation, however, the range of LPC (r^2^ = 0.385, *P* = 0.014) significantly increased (S4 Fig). Additionally, some traits deviated significantly from null expectations in specific elevational plots. For instance, a nonrandom pattern was detected for SLA and LPC at low elevation with high species richness (S4 and S5 Figs), a possible indication that accounting for intraspecific variation may improve the detection of environmental filtering, however, the detection of environmental filtering was not improved by accounting for intraspecific variability for SLA and LPC at low elevations (S3 and S4 Figs).

### Effect of intraspecific variability on limiting similarity

**Fig 3:**
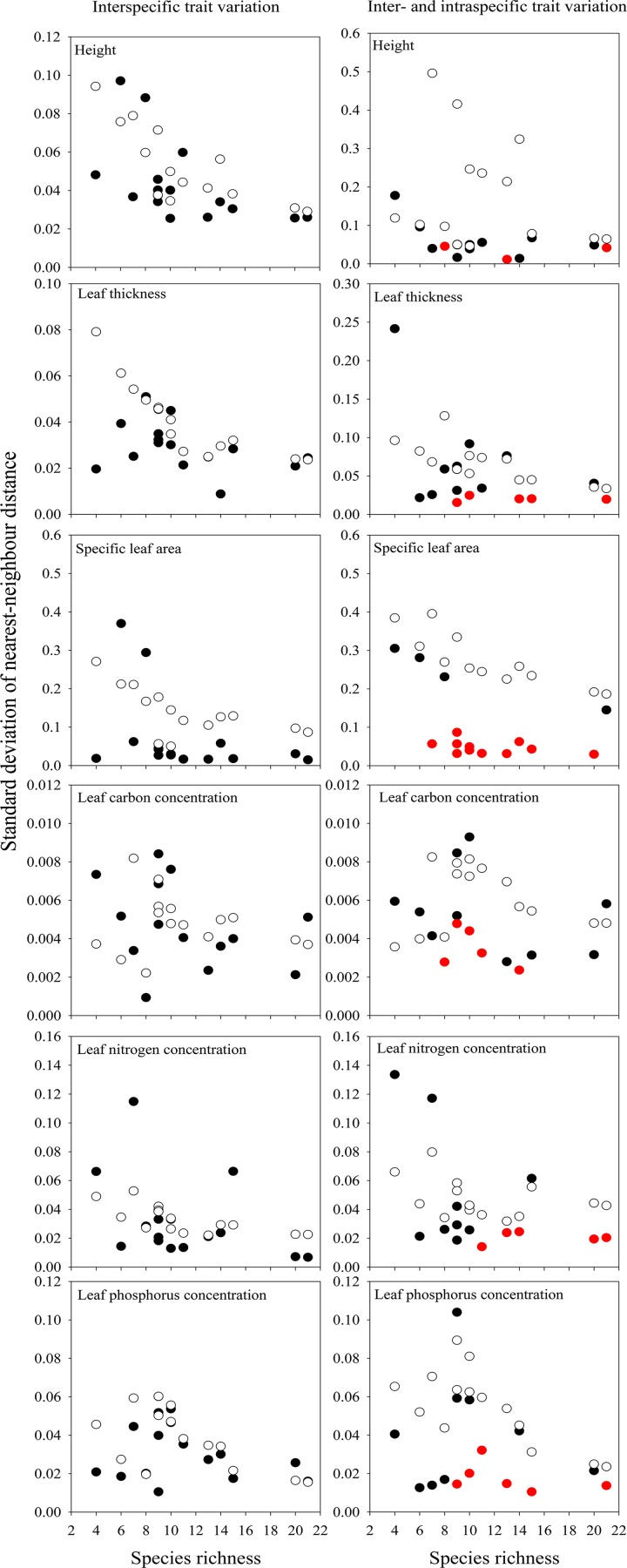
The importance of intraspecific trait variability in limiting similarity. Standard deviation of nearest-neighbour distance of functional traits along species richness using restricted null model, by accounting for intraspecific variability or not. Open circles indicate mean standard deviation of nearest-neighbour distance of trait values in random communities, solid circles indicate observed trait standard deviation of nearest-neighbour distance values, red circles indicate significant reductions in standard deviation of nearest-neighbour distance trait compared to a null model.

We found strong evidence that limiting similarity acted on all functional traits in most plots. The observed standard deviation of nearest-neighbor distance for all traits in at least 11 out of 15 plots were significantly smaller than expectations (Table 2), and the observed values significantly decreased with increasing species richness (*P*<0.05) (S6 Fig). Height, SLA and LNC appeared to be driving patterns in high species richness plots (S6 Fig). By contrast, LPC divergence plays a vital role in low richness plots at low elevations. Accounting for intraspecific variation revealed stronger evidence of limiting similarity acting on height, SLA and LPC than tests using only interspecific trait values, specifically, a greater number of significant plots were detected by accounting for intraspecific variation in height (3 vs. 0), SLA (11 vs. 4) and LPC (4 vs. 1) (Table 2). Nevertheless, intraspecific variability also improved the detection of significant trait divergence when using the seed dispersal limitation null model. For example, more plots were smaller than expectations when intraspecific variability was taken into account (Fig 3).

### Intra- and interspecific variability of CWM along different environmental axes

Climatic variables and soil water content significantly influenced CWM of height, SLA, LCC and LNC (Table 3). For instance, soil water content explained 26% to 48% of the total variance in height, SLA, LCC and LNC. Furthermore, AP and AMAT explained 66% and 74% of the variation in CWM of LCC values, respectively. However, for soil nutrients, the percent of variance explained in CWM of height, SLA, LCC and LNC was lower than soil water content and climatic variables. Further, variation in leaf thickness and LPC was not explained by any of the environmental variables. Models including multiple predictors improved the explanatory power of the relationship between most community mean traits in response to soil and climatic variables (S3 Table).

**Table 3:** General linear model regressions of community-weighted mean traits values by environmental variables.

Environmental variables	Height			Leaf thickness			SLA			LCC			LNC			LPC		
	*R*^*2*^	*F*	*P*	*R*^*2*^	*F*	*P*	*R*^*2*^	*F*	*P*	*R*^*2*^	*F*	*P*	*R*^*2*^	*F*	*P*	*R*^*2*^	*F*	*P*
SoilPC1	0.02	0.29	0.60	0.02	0.28	0.61	0.00	0.05	0.83	0.12	1.72	0.21	0.00	0.05	0.83	0.02	0.28	0.61
SoilPC2	0.00	0.02	0.90	0.03	0.38	0.55	0.30	5.52	**0.04**	0.17	2.70	0.12	0.24	4.03	0.07	0.03	0.35	0.57
SoilPC3	0.36	7.38	**0.02**	0.06	0.77	0.40	0.30	5.50	**0.04**	0.19	3.06	0.10	0.09	1.34	0.27	0.00	0.01	0.90
SMAT	0.37	7.76	**0.02**	0.03	0.39	0.54	0.54	15.02	**0.002**	0.64	22.96	**<0.001**	0.15	2.21	0.16	0.08	1.18	0.30
Soil water content	0.26	4.57	**0.05**	0.00	0.00	0.94	0.48	12.22	**0.004**	0.41	9.10	**0.01**	0.26	4.68	**0.05**	0.01	0.10	0.76
AP	0.11	1.64	0.22	0.08	1.17	0.30	0.44	10.10	**0.01**	0.66	25.47	**<0.001**	0.19	2.96	0.11	0.08	1.18	0.30
AMAT	0.14	2.04	0.18	0.11	1.55	0.24	0.38	7.99	**0.01**	0.74	36.45	**<0.001**	0.16	2.53	0.14	0.08	1.08	0.32

Values in bold are significant (*P*< 0.05). SoilPC1 = Soil PCA-axis 1; SoilPC2 = Soil PCA-axis 2; SoilPC3 = Soil PCA-axis 3; SMAT = soil mean annual temperature; AP = annual precipitation; AMAT = air mean annual temperature and soil water content was the March (dry season) water content.

**Fig 4:**
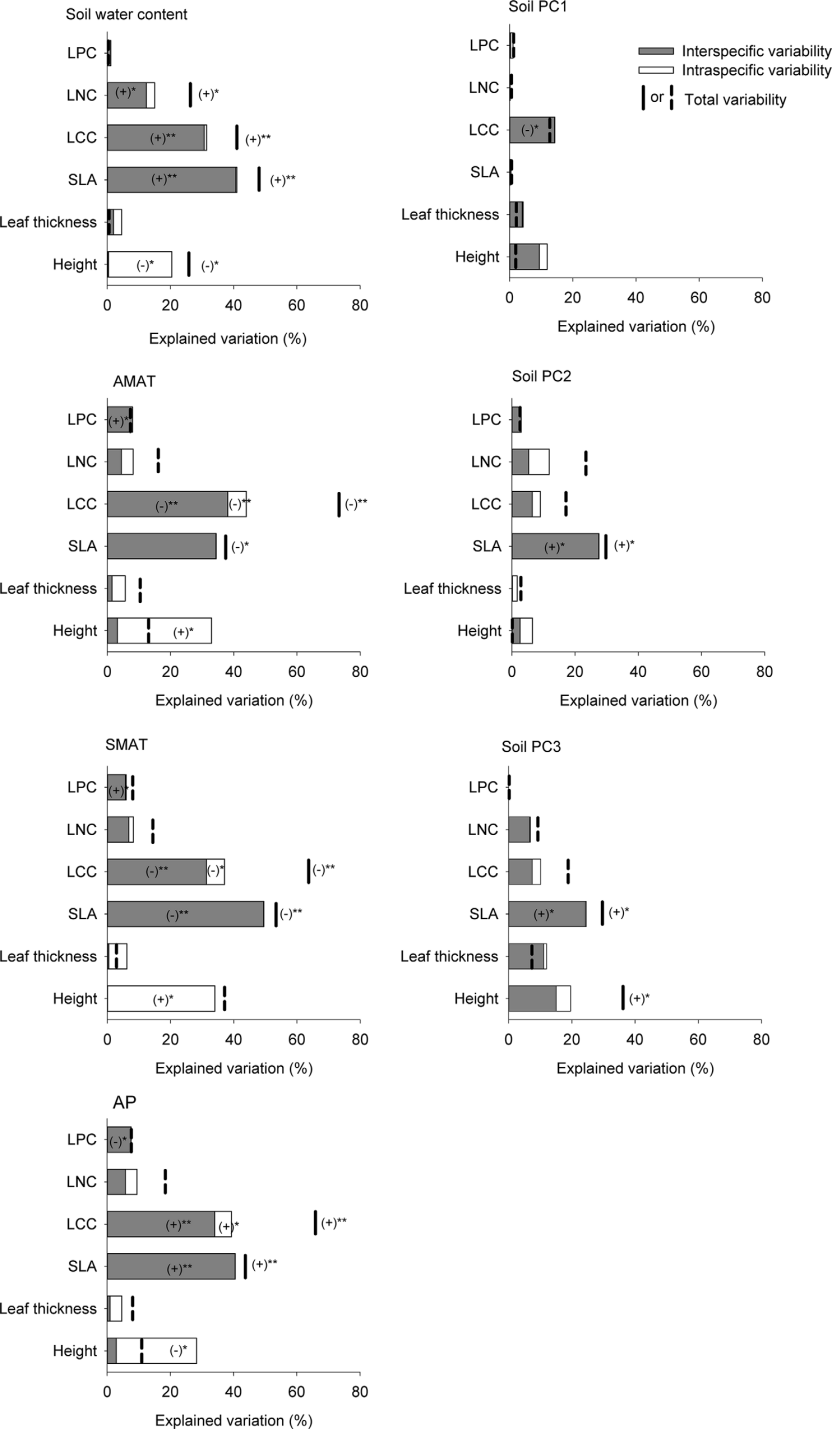
The relative contribution of interspecific, intraspecific and covariation effects to total variability of CWM trait values along different environmental axes. The signs (+ and -) and statistical significance (* *P*<0.05; ** *P*<0.01) of interspecific, intraspecific and total variability effects are showed on the figure when significant. A value of total variability that is higher than the sum of inter- and intraspecific variability indicates positive covariation, and a value of total variability that is lower than the sum of inter- and intraspecific variability indicates negative covariation. Solid lines indicate the significant relationship between total variability and environmental variable, and dash lines indicate non-significant relationship between total variability and environmental variable. Abbreviations: SLA, specific leaf area; LCC, leaf carbon concentration; LNC, leaf nitrogen concentration; LPC, leaf phosphorus concentration; AP, annual precipitation; AMAT, air mean annual temperature; SMAT, soil mean annual temperature; SoilPC1, Soil PCA-axis 1; Soil PC2, Soil PCA-axis 2; Soil PC2, Soil PCA-axis 2; SoilPC3, Soil PCA-axis 3.

The interspecific variation in SLA, LCC and LNC were all significantly correlated with soil water content. Climatic variables such as AMAT, SMAT and AP also explained a large amount of the variation in interspecific variation of SLA and LCC. However, only interspecific variation of LCC was significantly correlated with soil nutrients, and the explanatory power was smaller than climatic variables and soil water content (Fig 4).

Intraspecific variation of height was also explained by soil water content and climatic variables (Fig 4). However, the environmental axes under consideration here could not explain intraspecific variation in LNC and LPC, even though LNC and LPC have larger intraspecific variation than interspecific variation.

## Discussion

### The relative importance of intraspecific variability and species turnover

In previous trait-based community ecology studies, species were most frequently characterized by mean trait values only [4, 21]. However, recent studies have revealed the importance of intraspecific trait variation in determining community functional composition along environmental gradients [19, 23, 53, 54]. Despite this, how intraspecific variation influences such functional shifts is not well understood, especially in subalpine forests. Our results show that variation in SLA is largely driven by species turnover along elevational gradients, and the low contribution of intraspecific variation in SLA could reflect the dissimilarity between gymnosperm and angiosperm in high species turnover forests. In other words, the importance of intraspecific variation is relative to the magnitude of the differences in mean trait values [55]. Thus, we can expect a greater importance for intraspecific variability in SLA for forests dominated by relatively closely related species (e.g., oak forests). Furthermore, abundance-weighted community functional structure could also be influenced by the traits of dominant species. The observed increase in SLA values along the elevational gradient was undoubtedly due to species turnover from coniferous to evergreen broad-leaf forest. Meanwhile, leaves with larger SLA are constrained to having higher LCC investment at high elevation.

It is normally assumed that interspecific variation is the main contributor to functional trait change in high species turnover communities along steep elevational gradients [23]. However, in the present study, intraspecific variability of plant height, LNC and LPC accounts for 76.3%, 49.3% and 68.7% variation in functional turnover, respectively. Unlike leaf physical traits, plant height is usually associated with climatic and topographic variables, while LNC and LPC are associated with photosynthetic rate and nutrient cycling [1, 46, 56]. High variability in plant height and leaf chemical trait responses to stress or environmental gradients has been reported in previous studies [19, 28, 29, 31, 37]. This indicates that plant height and chemical traits are more plastic than leaf physical traits in subalpine forests. Our results also suggest that the relative contribution of intraspecific variation largely influences local functional composition.

### Role of intraspecific variability in environmental filtering

Environmental filtering, results in a certain range of trait values persisting in specific environmental conditions, has been suggested to drive community assembly [5, 15]. It has been reported that SLA decreases with increasing elevation, which reinforces the belief that environmental filtering is important at higher elevations [57]. In the present study, compared with null model, CWM of SLA significantly shifted toward the null model at low elevation, which implied that species are able to allocate more resources to leaves at low elevations than high elevations. For example, conifer species (*P*. *armandii* and *P*. *yunnanensis*) dominated at low elevations, which had low SLA. Additionally, the lower range of plant height at high elevation likely reflects convergence because of aboveground environmental stress, such as low temperature, atmospheric pressure, high wind velocity and radiation, and belowground soil water limitation [58]. Thus species with similar plant heights, such as *Quercus aquifolioides*, *Abies georgei* and *Picea likiangensis*, are the dominant species at high elevation in our study area. In contrast, the observed range of LPC values was less than predicted, and this convergence possibly reflects a response to disturbances like grazing and logging at low elevations, as human disturbance (grazing, logging, tourism development, etc.) occurs frequently at low elevations at Yulong Mountain [44].

Intraspecific trait variability has been considered in a limited number of community assembly studies, and findings reveal that measuring intraspecific variability greatly increases the detection of environmental filtering [7, 19, 32]. However, we found that the detection of environmental filtering did not improve after accounting for intraspecific trait variation. This might reflect that subalpine forest communities displayed stronger patterns of even spacing than convergence in functional traits. Our results did not correspond with previous studies [7, 19, 32], which indicated that environmental filtering is a widespread process influencing plant community assembly.

### Importance of intraspecific variability in limiting similarity

Some plant trait differences might result from more diverse groups of species that have adapted to very different habitat types. Such a result might also be related to more intense competition that drives fine-scale niche differences [17, 59, 60]. The observed standard deviation of nearest-neighbor distance of plant height, SLA, LCC and LPC were smaller than null expectations at low elevation thereby suggesting that the co-occurrence of more species could limit the similarity of functional traits to increase the even spacing of trait distance [9, 10, 61].

Quantifying intraspecific trait variability improved the likelihood of detecting limiting similarity. The divergence of traits within community (i.e. small SD_NND) suggests that forests may be assembled in such a way as to maximize resource utilization in order to reduce overlap of resource utilization [19, 32]. Intraspecific variability, through trait plasticity, promotes species coexistence through resource partitioning [17]. To improve the understanding of species coexistence in communities, we encourage the measurement of traits at multiple levels of biological organization, including at the population and/or individual level in future trait-based studies.

### Intra- and interspecific trait variability respond to different environmental axes

The relative importance of soil and climatic variables in driving both convergence and divergence of co-occurring functional traits has been demonstrated in previous studies [17, 60]. For woody species, temperature has been shown to correlate with leaf morphological traits such as SLA [1]. Our results revealed that climatic variables might influence the community-level average of SLA for subalpine canopy species more than soil nutrients. In addition, soil moisture was likely to be associated with tree traits [5, 7]. In the present study, available soil water content in the dry season may be a major limiting factor for community assembly in subalpine forests in Yulong Mountain. Precipitation is usually associated with leaf chemical traits [62]. However, we did not find evidence that leaf chemical traits were correlated with soil resources or climatic variables. This may reflect the fact that biotic factors, such as competition, which might affect leaf chemical trait variation, are more important than climatic factors in community assembly.

The relative importance of different environmental axes (e.g. climatic vs. non-climatic factors) to intraspecific variation varied among traits. Soil properties contribute to intraspecific variation in leaf traits [23, 54, 63]. Furthermore, it has been reported that a greater contribution of intraspecific variation in SLA and height occurs along non-climatic axes (e.g., soil properties, light) than along the main climatic axis for understory herbaceous species [35]. Plant height also may be modified by climatic variables (i.e. precipitation), age of succession, disturbance level and land use change [58, 64, 65]. In the present study, the intraspecific variation in plant height was more sensitive to climatic variables and soil water content changes than soil nutrients along elevational gradient in subalpine forest. Plant height was also shown to be highly responsive to soil resources such as available phosphorus in previous studies [24, 66, 67]. However, plant height was not associated with soil phosphorus in our study. Since species tend to use different nutrients to adapt to local ecological niches, the shifting pattern of available nutrients at different elevations could promote intraspecific trait variability [23, 68].

## Conclusions

Our results revealed that the contribution of intraspecific variability was even more substantial than interspecific variability for some traits, including plant height, LNC and LPC, despite the high level of species turnover along the elevational gradient in Yulong Mountain. We demonstrated that intraspecific trait variability improved the detection of community assembly processes (i.e. limiting similarity). We also observed trait convergence and divergence at specific elevations, and the detection of limiting similarity for most traits was significantly improved by accounting for intraspecific variability. Local functional trait plasticity appears to play an important role in driving processes of community assemblages along elevational gradients. Soil water content and climatic variables had a significant effect on most of traits except leaf thickness and LPC variation, whereas soil properties did not explain the trait variation. Our findings support the proposition that understanding species co-existence requires measures of population or individual level of trait variability. Both abiotic stress and biotic interactions together appear to drive the changes in community functional composition and influence intraspecific trait plasticity, especially in plant height and leaf chemical traits.

## Supporting Information

S1 Fig Principal component analysis of soil variables for all plots. (PDF)

S2 Fig Mantel test for the relation of species composition Sorensen pair-wise dissimilarity and change in elevation distance. (PDF)

S3 Fig Community weighted mean of functional traits along elevational gradient, by accounting for intraspecific variability or not. Solid black lines indicate a significant relationship; dashed black lines indicate regressions were statistically non-significant. Black points indicate communities that are not statistically different from random communities; red points indicate significant reductions in trait CWM compared to a null model; and blue points indicate significant increases in trait CWM compared to a null model.
(PDF)

S4 Fig Role of intraspecific trait variability in environmental filtering. Range of traits along elevational gradient both by considering intraspecific variability or not. Solid black lines indicate a significant relationship; dashed black lines indicate regressions were statistically non-significant. Black points indicate communities that are not statistically deviation from random communities; red points indicate significant reductions in trait range compared to a null model.
(PDF)

S5 Fig Range of traits along species richness both by considering intraspecific variability or not. Open circles indicate mean the range of trait values in random communities, solid circles indicate observed trait range values, red circles indicate significant reductions in range trait compared to a null model.
(PDF)

S6 Fig Importance of intraspecific trait variability in limiting similarity. **Standard deviation of nearest-neighbour distance of traits along species richness both by considering intraspecific variability or not.** Open circles indicate mean standard deviation of nearest-neighbour distance of trait values in random communities, solid circles indicate observed trait standard deviation of nearest-neighbour distance values, red circles indicate significant reductions in standard deviation of nearest-neighbour distance trait compared to a null model.
(PDF)

S1 Table Species checklist and traits data (mean±sd) for each species based on plot level in this study. Species recorded in only one plot have no value for variability.
(PDF)

S2 Table The plot information along elevational gradient of Yulong Mountain. (PDF)

S3 Table Relationships between CWM trait values and environmental variables (including soil and climatic variables). Multiple general linear model analysis was performed to select best multiple combinations of environmental variables which could predict community functional composition. Model being selected with lowest Akaike information criteria (AIC). Direction of correlation indicated by positive (+) and negative (–) signs. SWC = March soil water content; SoilPC1 = Soil PCA-axis 1; SoilPC2 = Soil PCA-axis 2; SoilPC3 = Soil PCA-axis 3; AP = annual precipitation; AMAT = air mean annual temperature; SMAT = Soil mean annual temperature.
(PDF)

## References

[1] Wright IJ, Reich PB, Westoby M, Ackerly DD, Baruch Z, Bongers F, et al (2004) The worldwide leaf economics spectrum. Nature 428: 821–827. 15103368 10.1038/nature02403

[2] Hooper DU, Chapin FS, Ewel JJ, Hector A, Inchausti P, Lavorel S, et al (2005) Effects of biodiversity on ecosystem functioning: A consensus of current knowledge. Ecological Monographs 75: 3–35.

[3] Grime JP (2006) Trait convergence and trait divergence in herbaceous plant communities: Mechanisms and consequences. Journal of Vegetation Science 17: 255–260.

[4] McGill BJ, Enquist BJ, Weiher E, Westoby M (2006) Rebuilding community ecology from functional traits. Trends in Ecology & Evolution 21: 178–185. 16701083 10.1016/j.tree.2006.02.002

[5] Ackerly DD, Cornwell W (2007) A trait-based approach to community assembly: partitioning of species trait values into within-and among-community components. Ecology Letters 10: 135–145. 17257101 10.1111/j.1461-0248.2006.01006.x

[6] Laughlin DC (2014) Applying trait- based models to achieve functional targets for theory‐driven ecological restoration. Ecology Letters 17: 771–784. 10.1111/ele.12288 24766299

[7] Cornwell WK, Ackerly DD (2009) Community assembly and shifts in plant trait distributions across an environmental gradient in coastal California. Ecological Monographs 79: 109–126.

[8] Webb CT, Hoeting JA, Ames GM, Pyne MI, Poff NL (2010) A structured and dynamic framework to advance traits-based theory and prediction in ecology. Ecology Letters 13: 267–283. 10.1111/j.1461-0248.2010.01444.x 20455917

[9] Kraft NJB, Valencia R, Ackerly DD (2008) Functional traits and niche-based tree community assembly in an Amazonian forest. Science 322: 580–582. 10.1126/science.1160662 18948539

[10] Kraft NJ, Ackerly DD (2010) Functional trait and phylogenetic tests of community assembly across spatial scales in an Amazonian forest. Ecological Monographs 80: 401–422.

[11] Messier J, McGill BJ, Lechowicz MJ (2010) How do traits vary across ecological scales? A case for trait‐based ecology. Ecology Letters 13: 838–848. 10.1111/j.1461-0248.2010.01476.x 20482582

[12] Baraloto C, Hardy OJ, Paine CET, Dexter KG, Cruaud C, Dunning LT, et al (2012) Using functional traits and phylogenetic trees to examine the assembly of tropical tree communities. Journal of Ecology 100: 690–701.

[13] Carmona CP, Rota C, Azcárate FM, Peco B (2014) More for less: sampling strategies of plant functional traits across local environmental gradients. Functional Ecology 29: 579–588.

[14] Kraft NJ, Adler PB, Godoy O, James EC, Fuller S, Levine JM (2015) Community assembly, coexistence and the environmental filtering metaphor. Functional Ecology 29: 592–599.

[15] Mayfield MM, Levine JM (2010) Opposing effects of competitive exclusion on the phylogenetic structure of communities. Ecology Letters 13: 1085–1093. 10.1111/j.1461-0248.2010.01509.x 20576030

[16] Gerhold P, Cahill JF, Winter M, Bartish IV, Prinzing A (2015) Phylogenetic patterns are not proxies of community assembly mechanisms (they are far better). Functional Ecology 29: 600–614.

[17] Pillar VD, Duarte LDS, Sosinski EE, Joner F (2009) Discriminating trait-convergence and trait-divergence assembly patterns in ecological community gradients. Journal of Vegetation Science 20: 334–348.

[18] Adler PB, Fajardo A, Kleinhesselink AR, Kraft NJ (2013) Trait‐based tests of coexistence mechanisms. Ecology Letters 16: 1294–1306. 10.1111/ele.12157 23910482

[19] Jung V, Violle C, Mondy C, Hoffmann L, Muller S (2010) Intraspecific variability and trait-based community assembly. Journal of Ecology 98: 1134–1140.

[20] Swenson N G, Anglada-Cordero P, Barone JA (2011) Deterministic tropical tree community turnover: evidence from patterns of functional beta diversity along an elevational gradient. Proceedings of the Royal Society of London B: Biological Sciences 278: 877–884. 10.1098/rspb.2010.1369 PMC3049044 20861048

[21] Garnier E, Laurent G, Bellmann A, Debain S, Berthelier P, Ducout B, et al (2001) Consistency of species ranking based on functional leaf traits. New Phytologist 152: 69–83. 10.1046/j.0028-646x.2001.00239.x 35974476

[22] Lepš J, de Bello F, Šmilauer P, Doležal J (2011) Community trait response to environment: disentangling species turnover vs intraspecific trait variability effects. Ecography 34: 856–863.

[23] Kichenin E, Wardle DA, Peltzer DA, Morse CW, Freschet GT (2013) Contrasting effects of plant inter‐and intraspecific variation on community‐level trait measures along an environmental gradient. Functional Ecology 27: 1254–1261.

[24] Siefert A, Fridley JD, Ritchie ME (2014) Community functional responses to soil and climate at multiple spatial scales: When does intraspecific variation matter? PLoS ONE 9: e111189 10.1371/journal.pone.0111189 25329794 PMC4203824

[25] Albert CH, Grassein F, Schurr FM, Vieilledent G, Violle C (2011) When and how should intraspecific variability be considered in trait-based plant ecology? Perspectives in Plant Ecology, Evolution and Systematics 13: 217–225.

[26] Bolnick DI, Amarasekare P, Araújo MS, Bürger R, Levine JM, Novak M, et al (2011) Why intraspecific trait variation matters in community ecology. Trends in Ecology & Evolution 26: 183–192. 21367482 10.1016/j.tree.2011.01.009 PMC3088364

[27] Violle C, Enquist BJ, McGill BJ, Jiang L, Albert CH, Hulshof C, et al (2012) The return of the variance: intraspecific variability in community ecology. Trends in Ecology & Evolution 27: 244–252. 22244797 10.1016/j.tree.2011.11.014

[28] Auger S, Shipley B (2013) Inter‐specific and intra‐specific trait variation along short environmental gradients in an old‐growth temperate forest. Journal of Vegetation Science 24: 419–428.

[29] Jung V, Albert CH, Violle C, Kunstler G, Loucougaray G, Spiegelberger T (2014) Intraspecific trait variability mediates the response of subalpine grassland communities to extreme drought events. Journal of Ecology 102: 45–53.

[30] Carlucci MB, Debastiani VJ, Pillar VD, Duarte LD (2015) Between‐and within‐species trait variability and the assembly of sapling communities in forest patches. Journal of Vegetation Science 26: 21–31.

[31] Siefert A., Violle C, Chalmandrier L, Albert CH, Taudiere A, Fajardo A, et al (2015) A global meta‐analysis of the relative extent of intraspecific trait variation in plant communities. Ecology Letters 18: 1406–1419. 10.1111/ele.12508 26415616

[32] Siefert A (2012) Incorporating intraspecific variation in tests of trait-based community assembly. Oecologia 170: 767–775. 10.1007/s00442-012-2351-7 22580640

[33] Albert CH, Thuiller W, Yoccoz NG, Douzet R, Aubert S, Lavorel S (2010) A multi‐trait approach reveals the structure and the relative importance of intra‐vs. interspecific variability in plant traits. Functional Ecology 24: 1192–1201.

[34] Mason NW, Richardson SJ, Peltzer DA, de Bello F, Wardle DA, Allen RB (2012) Changes in coexistence mechanisms along a long‐term soil chronosequence revealed by functional trait diversity. Journal of Ecology 100: 678–689.

[35] Lajoie G, Vellend M (2015) Understanding context dependence in the contribution of intraspecific variation to community trait-environment matching. Ecology 96: 2912–2922. 27070011 10.1890/15-0156.1

[36] Plourde BT, Boukili VK, Chazdon RL (2014) Radial changes in wood specific gravity of tropical trees: inter‐and intraspecific variation during secondary succession. Functional Ecology 29: 111–120.

[37] Kang M, Chang SX, Yan ER, Wang XH (2014) Trait variability differs between leaf and wood tissues across ecological scales in subtropical forests. Journal of Vegetation Science 25: 703–714.

[38] Nogués-Bravo D, Araújo M, Romdal T, Rahbek C (2008) Scale effects and human impact on the elevational species richness gradients. Nature 453: 216–219. 10.1038/nature06812 18464741

[39] Song XY, Yao YF, Wortley A, Paudayal K, Yang SH, Li CS, et al (2012) Holocene vegetation and climate history at Haligu on the Jade Dragon snow mountain, Yunnan, SW China. Climatic Change 113: 841–866

[40] Yan B, Zhang J, Liu Y, Li Z, Huang X, Yang W, et al (2012) Trait assembly of woody plants in communities across sub‐alpine gradients: Identifying the role of limiting similarity. Journal of Vegetation Science 23: 698–708.

[41] Myers N, Mittermeier RA, Mittermeier CG, Da Fonseca GA, Kent J (2000) Biodiversity hotspots for conservation priorities. Nature 403: 853–858. 10706275 10.1038/35002501

[42] Li X, Walker D (1986) The plant geography of Yunnan Province, southwest China.Journal of Biogeography 13: 367–397.

[43] Zhang RZ, Zheng D, Yang QY, Liu Y H (1997) Physical geography of Hengduan Mountains Science Press, Beijing.

[44] Feng J, Wang X, Xu C, Yang Y, Fang J (2006). Altitudinal patterns of plant species diversity and community structure on Yulong Mountains, Yunnan. China. Journal of Mountain Science 24: 110–116.

[45] Wright IJ, Reich PB, Cornelissen JH, Falster DS, Garnier E, Hikosaka K, et al (2005) Assessing the generality of global leaf trait relationships. New Phytologist 166: 485–496. 15819912 10.1111/j.1469-8137.2005.01349.x

[46] Cornelissen J, Lavorel S, Garnier E, Diaz S, Buchmann N, Gurvich D, et al (2003) A handbook of protocols for standardised and easy measurement of plant functional traits worldwide. Australian Journal of Botany 51: 335–380.

[47] Abràmoff MD, Magalhães PJ, Ram SJ (2004) Image processing with ImageJ. Biophotonics International 11: 36–43.

[48] Turner MG, Tinker DB, Romme WH, Kashian DM, Litton CM (2004) Landscape patterns of sapling density, leaf area, and aboveground net primary production in postfire lodgepole pine forests, Yellowstone National Park (USA). Ecosystems 7: 751–775.

[49] Bland JM, Altman DG (1996) Transformations, means, and confidence intervals. British Medical Journal 312: 1079 8616417 10.1136/bmj.312.7038.1079 PMC2350916

[50] Kumordzi BB, Wardle DA, Freschet GT (2015) Plant assemblages do not respond homogenously to local variation in environmental conditions: functional responses differ with species identity and abundance. Journal of Vegetation Science 26: 32–45.

[51] Gotelli NJ, Graves GR (1996) Null models in ecology Smithsonian Institution Press, Washington, DC, USA.

[52] R Core Team. R: A Language and Environment for Statistical Computing. Vienna, Austria: R Foundation for Statistical Computing 2015: http://www.R-project.org.

[53] Pescador DS, de Bello F, Valladares F, Escudero A (2015) Plant trait variation along an altitudinal gradient in Mediterranean high Mountain grasslands: Controlling the species turnover effect. PLoS ONE 10: e0118876 10.1371/journal.pone.0118876 25774532 PMC4361585

[54] Jager MM, Richardson SJ, Bellingham PJ, Clearwater MJ, Laughlin DC, De Deyn G (2015) Soil fertility induces coordinated responses of multiple independent functional traits. Journal of Ecology 103: 374–385.

[55] Poorter H, Niinemets Ü, Poorter L, Wright IJ, Villar R (2009) Causes and consequences of variation in leaf mass per area (LMA): A meta‐analysis. New Phytologist 182: 565–588. 19434804 10.1111/j.1469-8137.2009.02830.x

[56] Pérez-Harguindeguy N, Díaz S, Garnier E, Lavorel S, Poorter H, Jaureguiberry P, et al (2013) New handbook for standardised measurement of plant functional traits worldwide. Australian Journal of Botany 61: 167–234.

[57] Hulshof CM, Violle C, Spasojevic MJ, McGill B, Damschen E, Harrison S et al (2013) Intra-specific and inter-specific variation in specific leaf area reveal the importance of abiotic and biotic drivers of species diversity across elevation and latitude. Journal of Vegetation Science 24: 921–931.

[58] Körner C (2007) The use of ‘altitude’in ecological research. Trends in Ecology & Evolution 22: 569–574. 17988759 10.1016/j.tree.2007.09.006

[59] Ackerly D, Schwilk D, Webb C (2006) Niche evolution and adaptive radiation: testing the order of trait divergence. Ecology 87: S50–S61. 16922302 10.1890/0012-9658(2006)87[50:neaart]2.0.co;2

[60] Read QD, Moorhead LC, Swenson NG, Bailey JK, Sanders NJ (2014) Convergent effects of elevation on functional leaf traits within and among species. Functional Ecology 28: 37–45.

[61] Lusk CH, Reich PB, Montgomery RA, Ackerly DD, Cavender-Bares J (2008) Why are evergreen leaves so contrary about shade? Trends in Ecology & Evolution 23: 299–303. 18439708 10.1016/j.tree.2008.02.006

[62] Onoda Y, Westoby M, Adler PB, Choong AM, Clissold FJ, Cornelissen JH, et al(2011) Global patterns of leaf mechanical properties. Ecology Letters 14: 301–312. 10.1111/j.1461-0248.2010.01582.x 21265976

[63] Pakeman RJ (2013) Intra-specific leaf trait variation: management and fertility matter more than the climate at continental scales. Folia Geobotanica 48: 355–371.

[64] Falster DS, Westoby M (2003) Plant height and evolutionary games. Trends in Ecology & Evolution 18: 337–343.

[65] Moles AT, Warton DI, Warman L, Swenson NG, Laffan SW, Zanne AE, et al (2009) Global patterns in plant height. Journal of Ecology 97: 923–932.

[66] Dantas VdL, Pausas JG, Batalha MA, de Paula Loiola P, Cianciaruso MV (2013) The role of fire in structuring trait variability in Neotropical savannas. Oecologia 171: 487–494. 10.1007/s00442-012-2431-8 22926723

[67] Gross N, Börger L, Soriano‐Morales SI, Bagousse‐Pinguet L, Quero JL, García‐Gómez M, et al (2013) Uncovering multiscale effects of aridity and biotic interactions on the functional structure of Mediterranean shrublands. Journal of Ecology 101: 637–649.

[68] McKane RB, Johnson LC, Shaver GR, Nadelhoffer KJ, Rastetter EB, Fry B, et al (2002) Resource-based niches provide a basis for plant species diversity and dominance in arctic tundra. Nature 415: 68–71. 11780117 10.1038/415068a

