# Supplementary figures and images for: Trait-Based Community Assembly along an Elevational Gradient in Subalpine Forests: Quantifying the Roles of Environmental Factors in Inter- and Intraspecific Variability
Source: PLoS One. 2016 May 18;11(5):e0155749. doi: 10.1371/journal.pone.0155749 (PMC4871540; doi:10.1371/journal.pone.0155749)

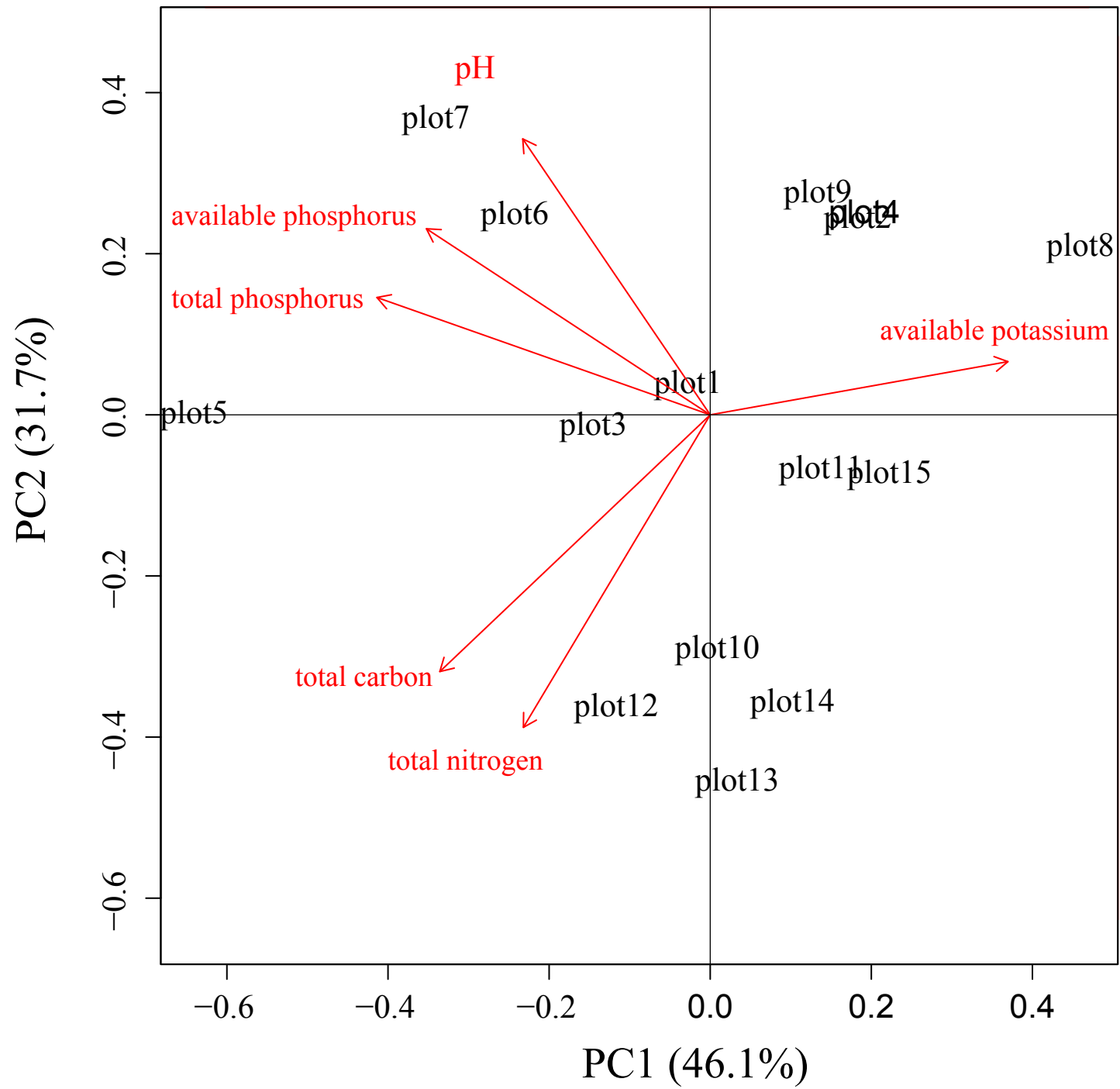

Supplement: S1 Fig — (PDF) [file pone.0155749.s001.pdf]

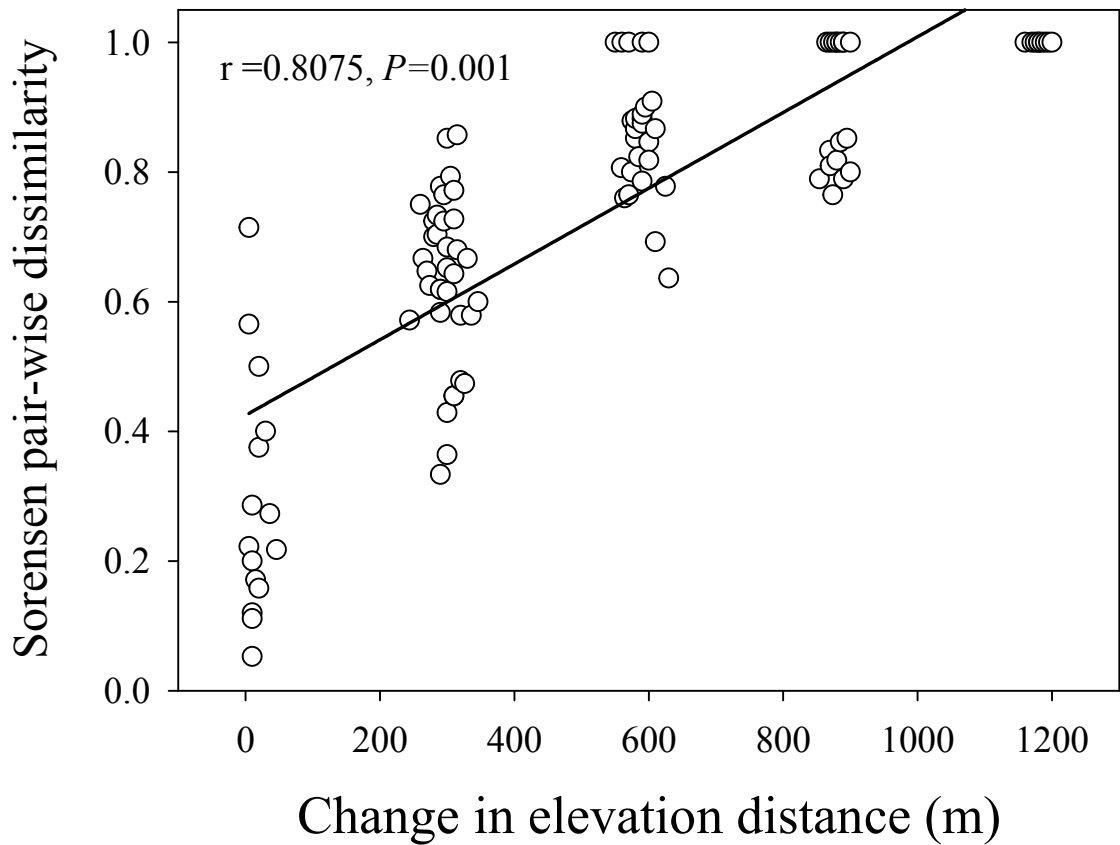

Supplement: S2 Fig — (PDF) [file pone.0155749.s002.pdf]

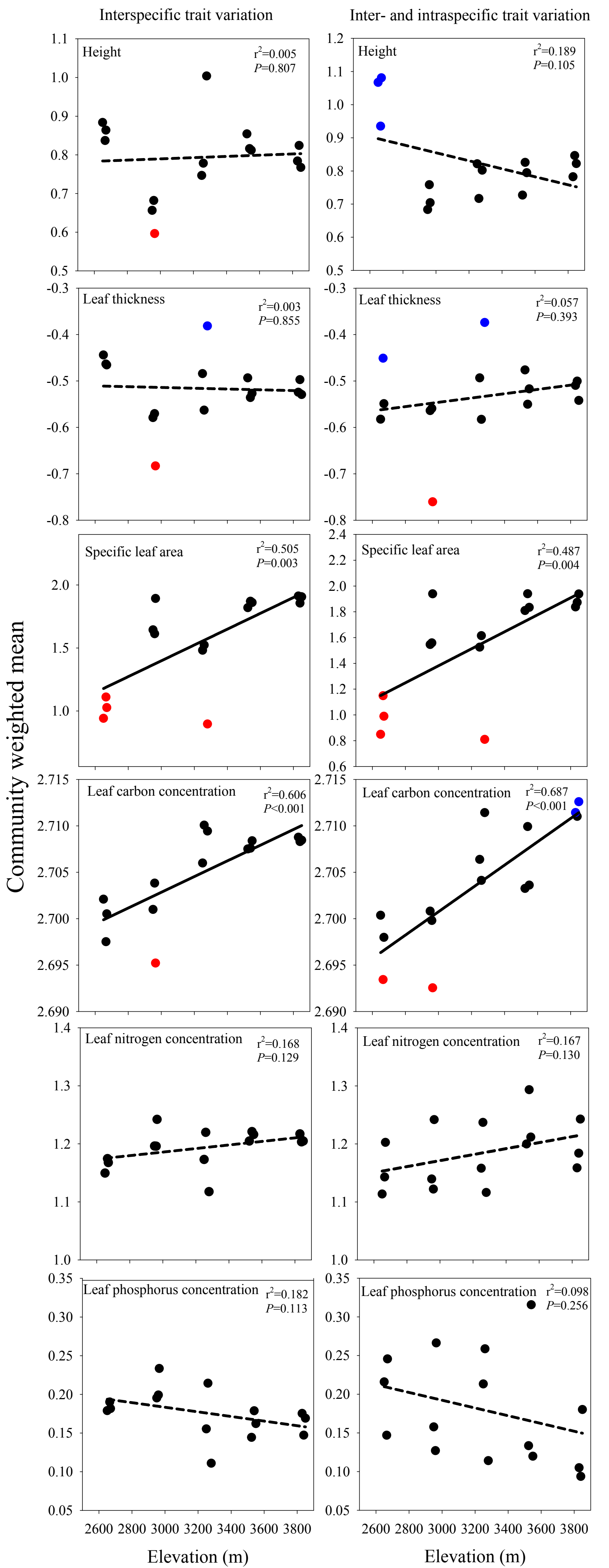

Supplement: S3 Fig — Solid black lines indicate a significant relationship; dashed black lines indicate regressions were statistically non-significant. Black points indicate communities that are not statistically different from random communities; red points indicate significant reductions in trait CWM compared to a null model; and blue points indicate significant increases in trait CWM compared to a null model. (PDF) [file pone.0155749.s003.pdf]

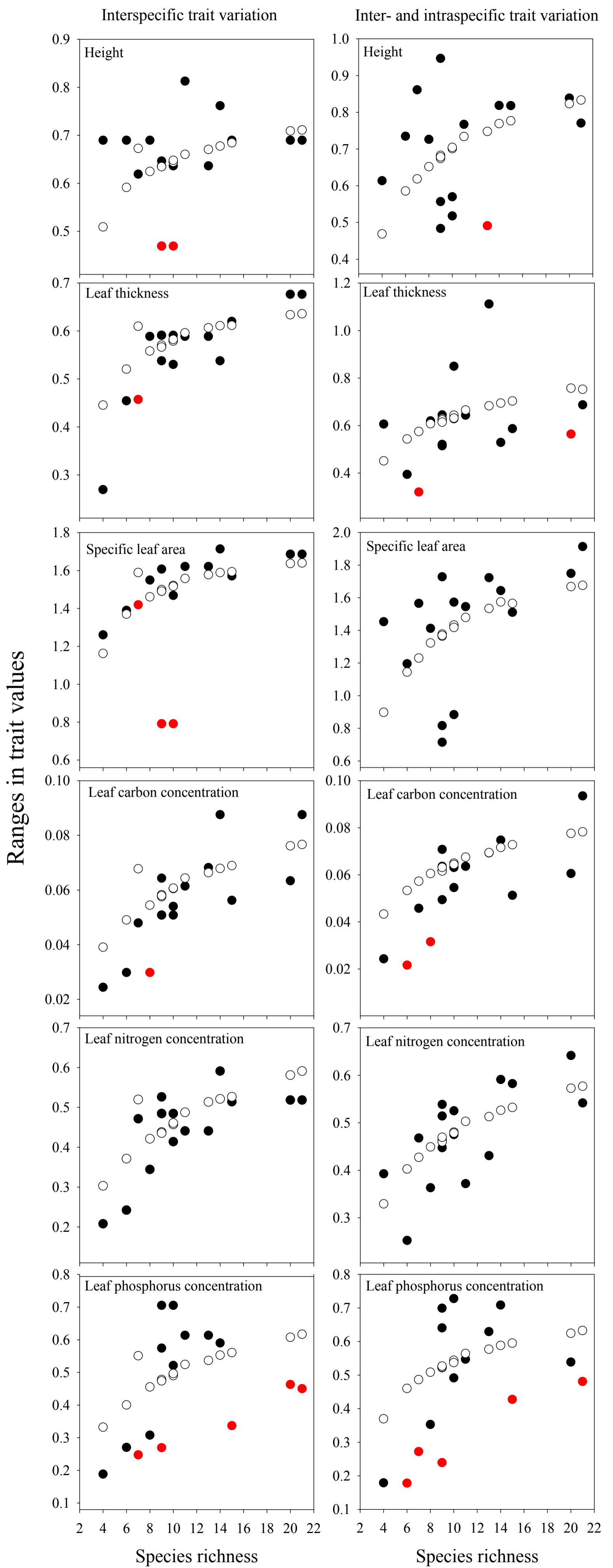

Supplement: S5 Fig — Open circles indicate mean the range of trait values in random communities, solid circles indicate observed trait range values, red circles indicate significant reductions in range trait compared to a null model. (PDF) [file pone.0155749.s005.pdf]

Interspecific trait variation

Inter- and intraspecific trait variation

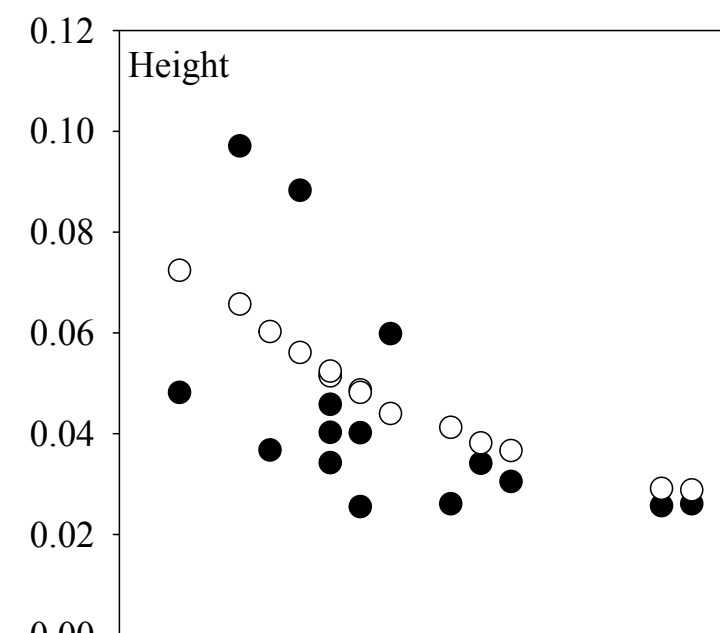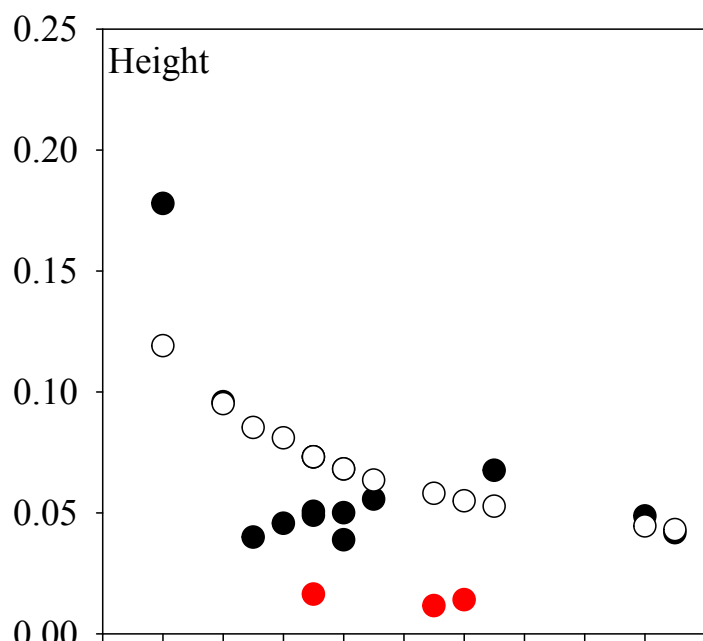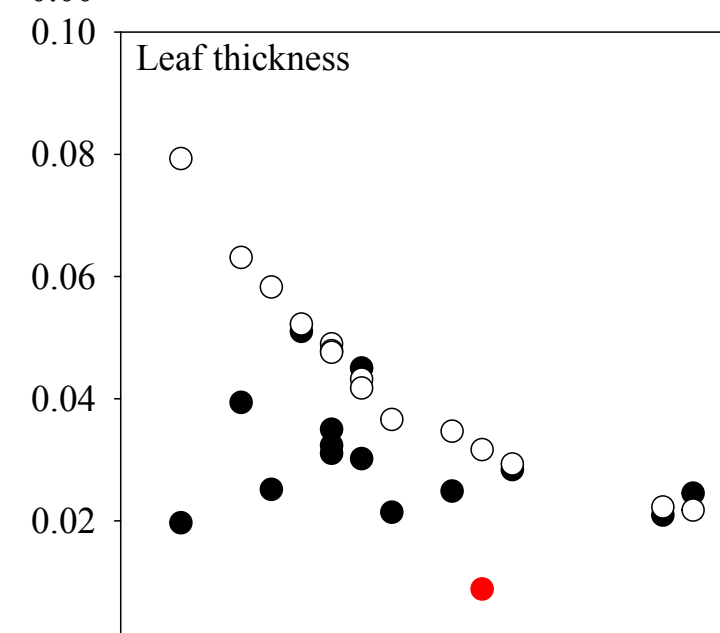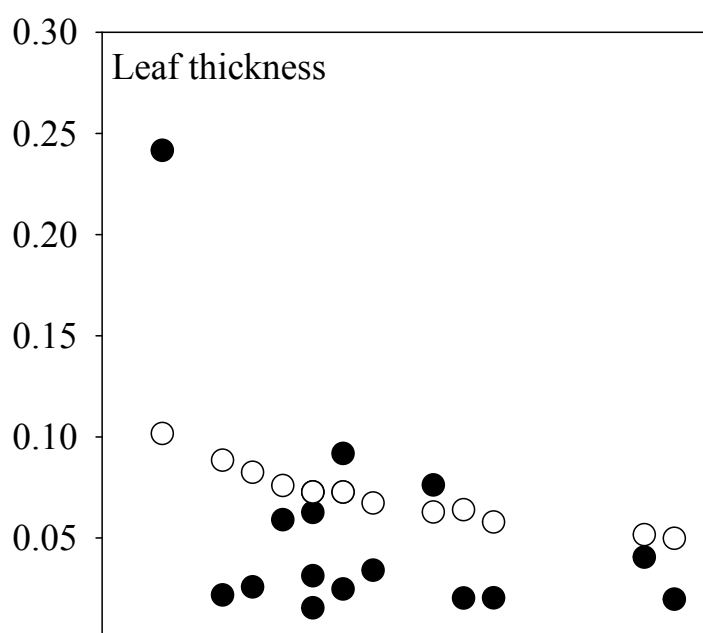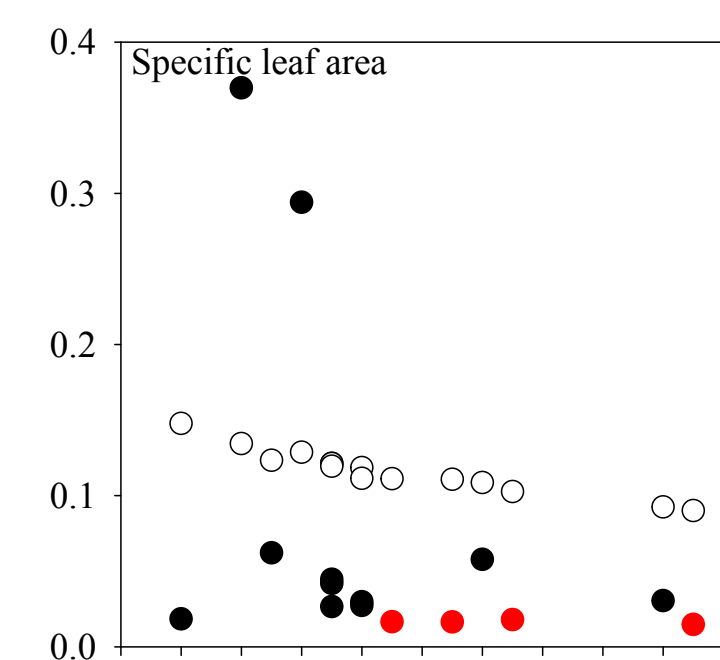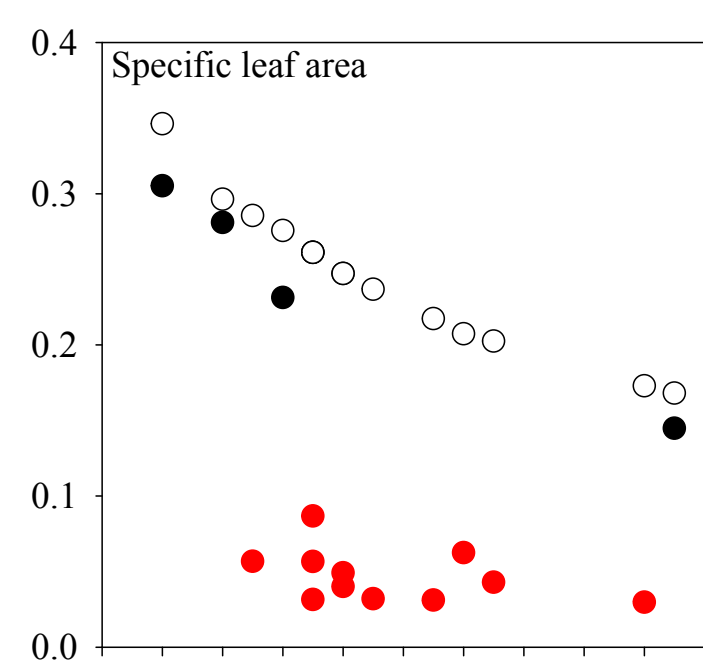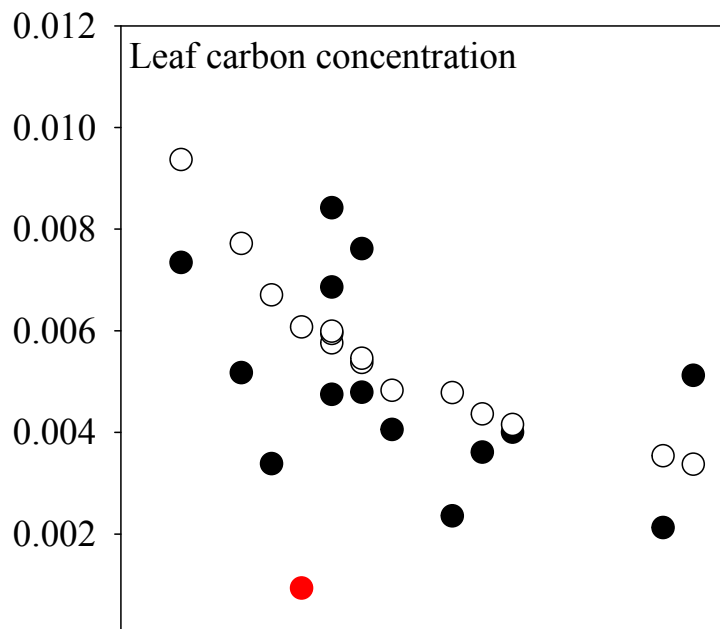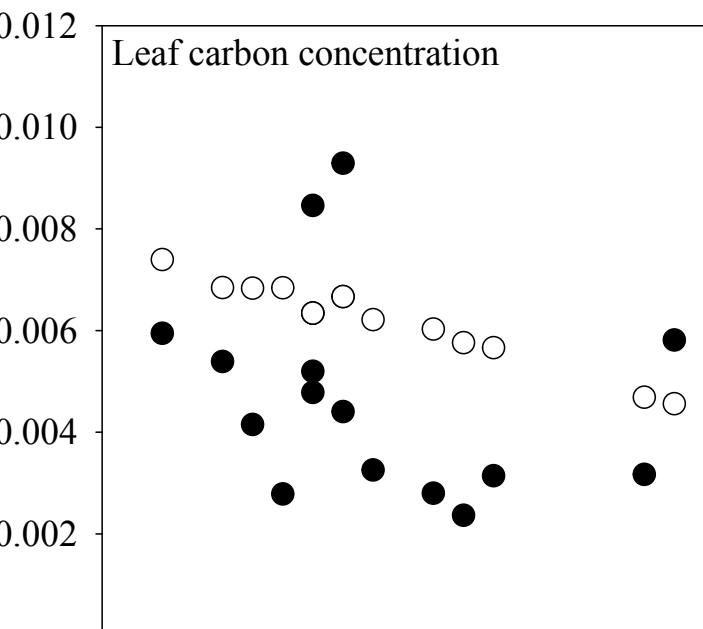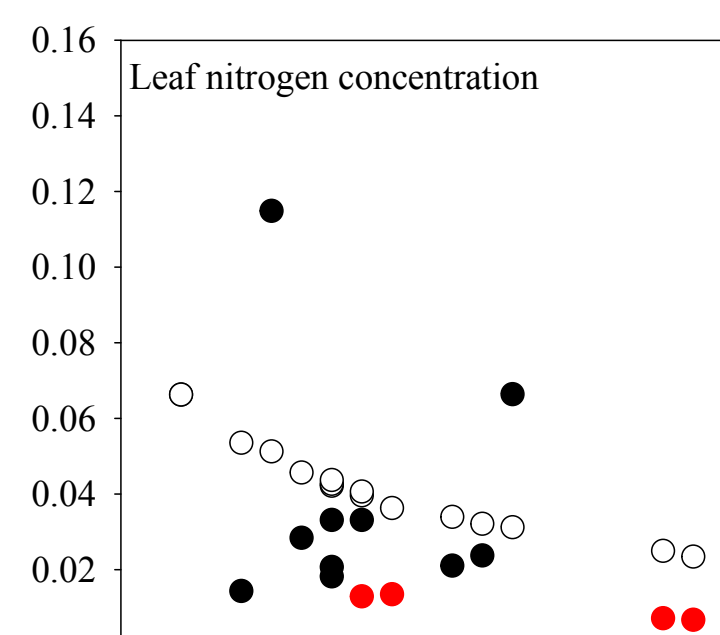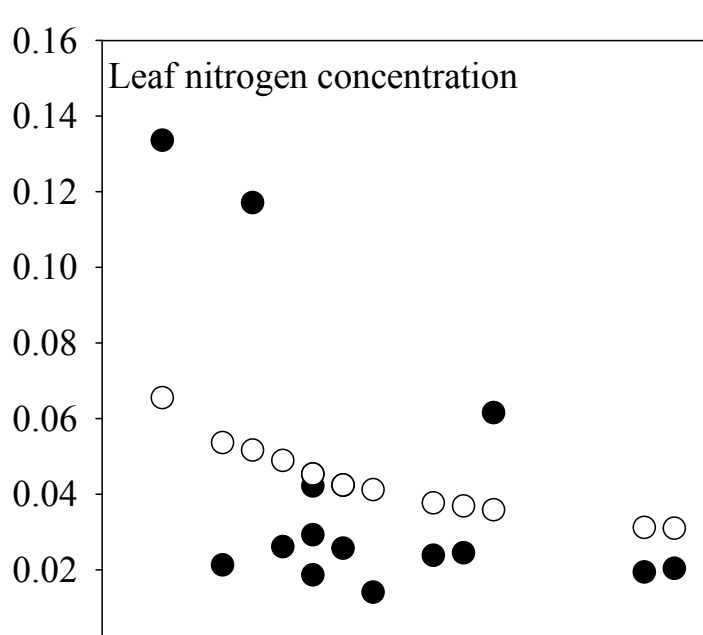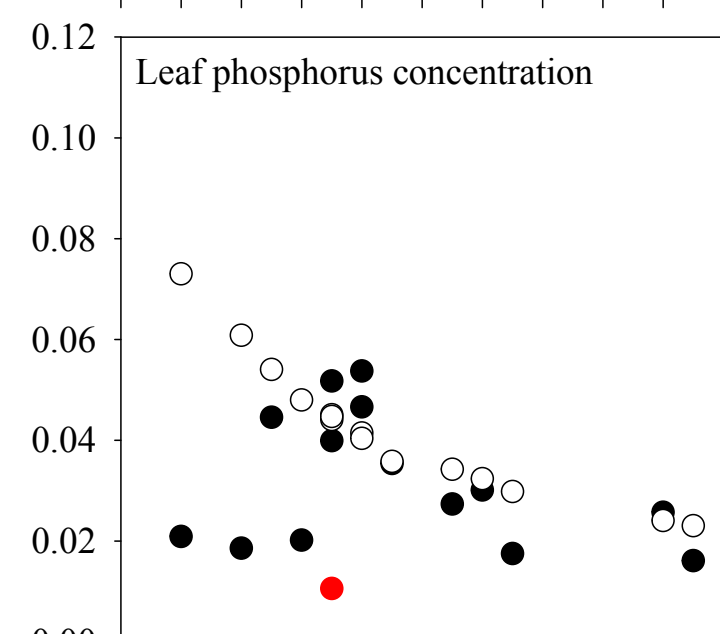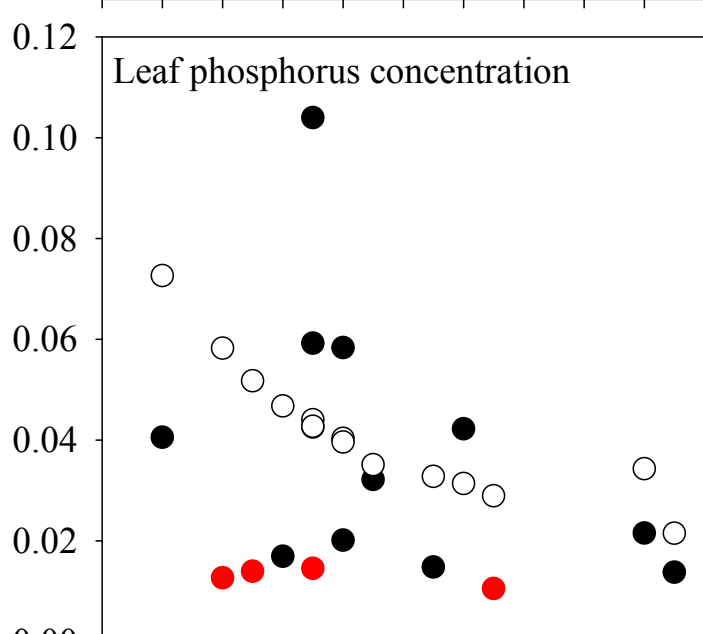

Supplement: S6 Fig — Standard deviation of nearest-neighbour distance of traits along species richness both by considering intraspecific variability or not. Open circles indicate mean standard deviation of nearest-neighbour distance of trait values in random communities, solid circles indicate observed trait standard deviation of nearest-neighbour distance values, red circles indicate significant reductions in standard deviation of nearest-neighbour distance trait compared to a null model. (PDF) [file pone.0155749.s006.pdf]
