# Supplementary material for: Trait-Based Community Assembly along an Elevational Gradient in Subalpine Forests: Quantifying the Roles of Environmental Factors in Inter- and Intraspecific Variability
Source: PLoS One. 2016 May 18;11(5):e0155749. doi: 10.1371/journal.pone.0155749 (PMC4871540; doi:10.1371/journal.pone.0155749)
Supplement: S4 Fig — Range of traits along elevational gradient both by considering intraspecific variability or not. Solid black lines indicate a significant relationship; dashed black lines indicate regressions were statistically non-significant. Black points indicate communities that are not statistically deviation from random communities; red points indicate significant reductions in trait range compared to a null model. (PDF) [file pone.0155749.s004.pdf]

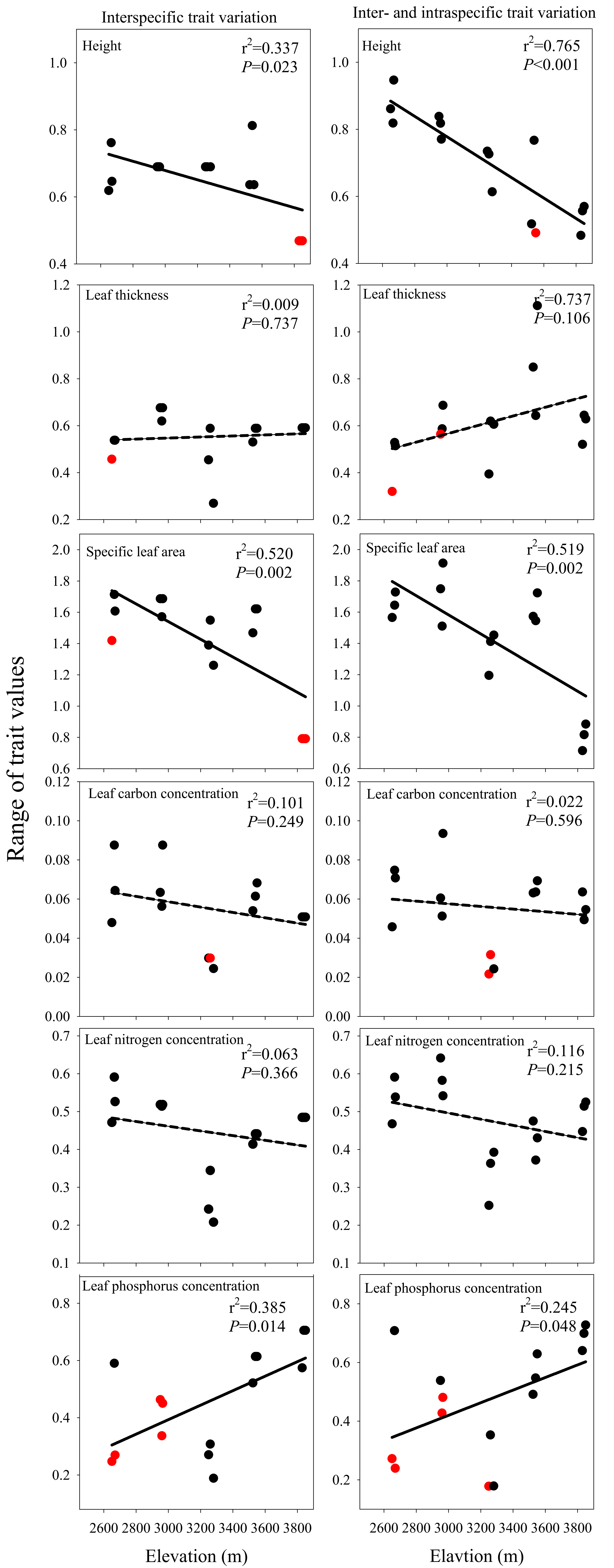

Interspecific trait variation
Inter- and intraspecific trait variation
Height
r²=0.337
P=0.023
Height
r²=0.765
P<0.001
Leaf thickness
r²=0.009
P=0.737
Leaf thickness
r²=0.737
P=0.106
Specific leaf area
r²=0.520
P=0.002
Specific leaf area
r²=0.519
P=0.002
Leaf carbon concentration
r²=0.101
P=0.249
Leaf carbon concentration
r²=0.022
P=0.596
Leaf nitrogen concentration
r²=0.063
P=0.366
Leaf nitrogen concentration
r²=0.116
P=0.215
Leaf phosphorus concentration
r²=0.385
P=0.014
Leaf phosphorus concentration
r²=0.245
P=0.048
Range of trait values
Elevation (m)
Elavtion (m)
