# Supplementary material for: Trait-Based Community Assembly along an Elevational Gradient in Subalpine Forests: Quantifying the Roles of Environmental Factors in Inter- and Intraspecific Variability
Source: PLoS One. 2016 May 18;11(5):e0155749. doi: 10.1371/journal.pone.0155749 (PMC4871540; doi:10.1371/journal.pone.0155749)
Supplement: S1 Table — Species recorded in only one plot have no value for variability. (PDF) [file pone.0155749.s007.pdf]

Category	Family	Species	Height (m)	Leaf thickness(mm)	SLA($cm^2.g^{-1}$)	LCC($g.kg^{-1}$)	LNC(g.kg-1)	LPC(g.kg-1)
Gymnosperm	Pinaceae	*Abies georgei*	5.73±1.93	0.396±0.072	48.853±9.286	512.3±9.3	10.43±0.31	1.05±0.15
Gymnosperm	Cupressaceae	*Juniperus formosana*	1.6	0.214	83.116	506	12.14	1.48
Gymnosperm	Pinaceae	*Larix potaninii var. australis*	7.9	0.189	123.696	512	18.63	1.79
Gymnosperm	Pinaceae	*Picea likiangensis*	5.03±1.17	0.422±0.005	43.320±3.984	505.7±0.6	9.43±0.14	0.93±0.01
Gymnosperm	Pinaceae	*Pinus armandii*	8.85±4.48	0.366±0.090	6.417±1.293	504.4±4.9	13.78±2.08	1.63±0.34
Gymnosperm	Pinaceae	*Pinus yunnanensis*	10.40±3.83	0.420±0.102	7.560±2.872	512.6±6.5	13.02±1.12	1.28±0.19
Gymnosperm	Pinaceae	*Tsuga dumosa*	6.5	0.376	70.609	508	13.55	1.87
Angiosperm	Sapindaceae	*Acer davidii*	4.37±0.57	0.137±0.04	187.280±61.061	467.0±8.5	17.79±0.68	2.33±0.51
Angiosperm	Berberidaceae	*Berberis levis*	3.07	0.384	69.678	470	11.54	1.76
Angiosperm	Berberidaceae	*Berberis pruinosa*	2.6	0.124	223.872	465	11.12	2.38
Angiosperm	Rosaceae	*Cerasus trichostoma*	5.2	0.104	206.955	445	20.76	3.6
Angiosperm	Coriariaceae	*Coriaria nepalensis*	2.63±0.67	0.200±0.005	135.734±6.825	474.5±4.9	23.57±0.69	1.20±0.11
Angiosperm	Rosaceae	*Cotoneaster dielsianus*	2.38±0.33	0.161±0.029	129.101±54.639	474.2±8.3	20.36±2.17	2.25±0.32
Angiosperm	Fabaceae	*Desmodium yunnanense*	3.14±0.76	0.089±0.029	311.287±86.395	473	42.97±7.76	2.72
Angiosperm	Caprifoliaceae	*Dipelta yunnanensis*	3.15	0.096	201.815	475	18.73	2.52
Angiosperm	Rosaceae	*Docynia delavayi*	2.9	0.189	128.166	491	16.74	3.02
Angiosperm	Elaeagnaceae	*Elaeagnus bockii*	2	0.206	122.446	482	37.39	1.48
Angiosperm	Elaeagnaceae	*Elaeagnus umbellata*	3.06±0.39	0.165±0.028	145.267±6.137	479.6±9.7	37.36±1.09	1.58±0.09
Angiosperm	Lamiaceae	*Elsholtzia fruticosa*	2	0.133	331.461	455	23.29	3.92
Angiosperm	Aquifoliaceae	*Ilex delavayi*	3.03±0.90	0.206±0.011	90.769±7.300	523.0±1.7	18.10±2.15	1.29±0.25
Angiosperm	Fabaceae	*Indigofera pendula*	3.12±0.74	0.101±0.013	238.789±50.446	450.3±9.9	42.55±2.00	2.17±0.37
Angiosperm	Oleaceae	*Ligustrum quihoui*	2.6	0.193	161.941	488	17.93	1.62
Angiosperm	Carpofoliaceae	*Lonicera lanceolata*	2.79±0.35	0.112±0.045	268.554	488.8±17.0	28.43±3.33	2.34±0.87
Angiosperm	Carpofoliaceae	*Lonicera setifera*	2.93±0.04	0.190±0.087	177.103±90.306	443.0±7.1	16.81±0.01	3.47
Angiosperm	Carpofoliaceae	*Lonicera tangutica*	3.40±0.42	0.152±0.020	210.211±4.531	470.5±7.7	25.91±0.21	4.7

Angiosperm	Ericaceae	*Lyonia ovalifolia*	3.48±0.70	0.152±0.011	175.271±27.687	504.0±1.7	21.87±2.57	2.05±0.23
Angiosperm	Ericaceae	*Lyonia villosa*	3.95±1.56	0.154±0.017	178.199±16.331	516.2±15.6	22.73±2.34	2.30±0.49
Angiosperm	Rosaceae	*Malus ombrophila*	3.61±1.29	0.105±0.021	220.173±24.689	483.0±17.5	22.93±5.19	2.77±0.95
Angiosperm	Rosaceae	*Malus yunnanensis*	3.6	0.154	138.961	472	14.93	2.22
Angiosperm	Saxifragaceae	*Philadelphus delavayi*	3.90±1.70	0.129±0.019	249.654±69.789	419.0±9.9	24.48±1.60	3.34±0.35
Angiosperm	Salicaceae	*Populus adenopoda*	5.50±2.57	0.148±0.019	148.632±30.113	492.2±15.3	23.15±2.61	2.29±0.46
Angiosperm	Rosaceae	*Prinsepia utilis*	1.8	0.196	229.055	427	45	4.65
Angiosperm	Rosaceae	*Pyracantha angustifolia*	2.5	0.191	82.671	459	12.62	1.01
Angiosperm	Rosaceae	*Pyrus pashia*	3.23±0.32	0.163±0.001	147.515±17.034	482.5±6.4	14.63	1.78±0.49
Angiosperm	Fagaceae	*Quercus aliena var. acutiserrata*	3.03±0.81	0.137±0.029	153.238±34.878	479.5±6.5	23.73±0.71	2.36±0.67
Angiosperm	Fagaceae	*Quercus aquifolioides*	8.49±1.64	0.387261905	51.653±4.718	508.0±5.1	15.15±1.93	1.20±0.20
Angiosperm	Fagaceae	*Quercus guyavifolia*	5.37±1.86	0.318125	69.239±22.440	504.7±2.5	14.36±2.22	1.40±0.23
Angiosperm	Fagaceae	*Quercus senescens*	2.58	0.2875	56.443	500	15.91	1.38
Angiosperm	Fagaceae	*Quercus spinosa*	3.61±1.49	0.223±0.064	85.492±9.724	499.7±6.8	14.35±0.60	1.28±0.11
Angiosperm	Rhamnaceae	*Rhamnus virgata*	2.58±0.50	0.122±0.024	259.689±6.385	442.0±9.9	29.75±2.42	1.80±0.45
Angiosperm	Ericaceae	*Rhododendron decorum*	2.34±0.56	0.251±0.069	112.833±37.560	484.1±5.5	15.32±4.14	1.49±0.54
Angiosperm	Ericaceae	*Rhododendron oreotrephes*	4.61±0.65	0.227±0.030	125.694±46.896	514.2±15.3	20.18±6.34	1.98±0.89
Angiosperm	Ericaceae	*Rhododendron racemosum*	2.13±0.39	0.226±0.067	116.855±33.216	510.7±3.6	21.015±3.55	1.98±0.34
Angiosperm	Ericaceae	*Rhododendron rubiginosum*	5.84±1.67	0.253±0.021	101.172±24.034	519.2±5.0	17.25±4.43	1.67±0.78
Angiosperm	Ericaceae	*Rhododendron traillianum*	6.11±0.33	0.358±0.036	79.835±14.561	515.3±1.5	15.57±2.47	1.43±0.30
Angiosperm	Ericaceae	*Rhododendron yunnanense*	3.19±0.48	0.171±0.014	149.751±24.545	501.7±3.3	19.40	2.04±0.23
Angiosperm	Grossulariaceae	*Ribes tenue*	3.30±1.13	0.132±0.023	249.105±30.981	454±12.7	25.28±0.29	4.30±0.01
Angiosperm	Rosaceae	*Rosa longicuspis*	2.6	0.176	127.890	471	12.99	1.67
Angiosperm	Salicaceae	*Salix balfouriana*	2.9	0.089	309.175	479	25.28	3.01
Angiosperm	Rosaceae	*Sorbus rehderiana*	4.28±0.83	0.124±0.036	188.555±32.304	461.8±5.0	27.03±3.25	3.48±0.67
Angiosperm	Rosaceae	*Spiraea lichiangensis*	2.7	0.130	160.065	447	21.82	4.23

Angiosperm	Rosaceae	*Stranvaesia davidiana*	2.58 ±0.92	0.166 ±0.006	82.952 ±8.889	496.7 ±6.7	13.52 ±1.50	1.86 ±0.39
Angiosperm	Adoxaceae	*Viburnum cylindricum*	3.15 ±0.93	0.284 ±0.044	87.096 ±20.697	510 ±2.4	12.75 ±2.52	1.30 ±0.363
Angiosperm	Adoxaceae	*Viburnum hupehense*	2.53 ±0.55	0.147 ±0.013	183.172 ±58.604	474.0 ±10.8	18.79 ±6.96	1.78 ±0.75
Angiosperm	Rutaceae	*Zanthoxylum bungeanum*	2.8	0.136	266.187	454	32.87	3.44
