# Supplementary material for: Trait-Based Community Assembly along an Elevational Gradient in Subalpine Forests: Quantifying the Roles of Environmental Factors in Inter- and Intraspecific Variability
Source: PLoS One. 2016 May 18;11(5):e0155749. doi: 10.1371/journal.pone.0155749 (PMC4871540; doi:10.1371/journal.pone.0155749)
Supplement: S2 Table — (PDF) [file pone.0155749.s008.pdf]

Plots	Elevation (m)	Tree density (individual / 0.1ha)	Species richness	Shannon index	Simpson index	Species evenness
1	2670	98	9	1.179	0.568	0.537
2	2650	89	7	0.932	0.467	0.479
3	2665	113	14	1.509	0.669	0.572
4	2960	356	15	1.940	0.817	0.716
5	2950	325	20	2.272	0.851	0.759
6	2965	451	21	2.463	0.879	0.809
7	3250	275	6	1.256	0.673	0.701
8	3260	341	8	1.269	0.651	0.610
9	3280	119	4	0.181	0.065	0.130
10	3524	300	10	1.042	0.549	0.452
11	3550	444	13	1.390	0.652	0.542
12	3540	420	11	1.450	0.671	0.583
13	3840	544	9	1.497	0.700	0.681
14	3850	476	10	1.761	0.807	0.765
15	3830	431	9	1.702	0.793	0.775
Total		4782	55			
