# Supplementary material for: Trait-Based Community Assembly along an Elevational Gradient in Subalpine Forests: Quantifying the Roles of Environmental Factors in Inter- and Intraspecific Variability
Source: PLoS One. 2016 May 18;11(5):e0155749. doi: 10.1371/journal.pone.0155749 (PMC4871540; doi:10.1371/journal.pone.0155749)
Supplement: S3 Table — Multiple general linear model analysis was performed to select best multiple combinations of environmental variables which could predict community functional composition. Model being selected with lowest Akaike information criteria (AIC). Direction of correlation indicated by positive (+) and negative (–) signs. SWC = March soil water content; SoilPC1 = Soil PCA-axis 1; SoilPC2 = Soil PCA-axis 2; SoilPC3 = Soil PCA-axis 3; AP = annual precipitation; AMAT = air mean annual temperature; SMAT = Soil mean annual temperature. (PDF) [file pone.0155749.s009.pdf]

Traits	Variables	Predictors	R^2
Height	Soil	SWC (+), SoilPC1 (-), SoilPC2 (+)	0.587
	Climatic	AMAT (-), SMAT (+)	0.789
Leaf thickness	Soil	ns	NA
	Climatic	AMAT (-), SMAT (+)	0.284
SLA	Soil	SWC(+), SoilPC1(+)	0.704
	Climatic	AMAT (+), SMAT (-), AP (+)	0.753
LCC	Soil	SoilPC1 (-), SoilPC2 (+), SoilPC3 (+)	0.479
	Climatic	AMAT (-), AP(-)	0.789
LNC	Soil	SWC (+)	0.265
	Climatic	AP (+)	0.083
LPC	Soil	ns	NA
	Climatic	ns	NA
